# Identification of Ndfip1 as a novel negative regulator for spatial memory formation associated with increased ubiquitination of Beclin 1 and PTEN

**DOI:** 10.1371/journal.pone.0283908

**Published:** 2023-04-06

**Authors:** Wei-Lun Hsu, Yun-Li Ma, Yan-Chu Chen, Yen-Chen Liu, Kuang-Min Cheng, Eminy H. Y. Lee

**Affiliations:** 1 Institute of Biomedical Sciences, Academia Sinica, Taipei, Taiwan; 2 Graduate Institute of Life Sciences, National Defense Medical Center, Taipei, Taiwan; Nathan S Kline Institute, UNITED STATES

## Abstract

Long-term memory formation requires *de novo* RNA and protein synthesis. By using the differential display-polymerase chain reaction strategy, we have presently identified the Nedd4 family interacting protein 1 (*Ndfip1*) cDNA fragment that is differentially expressed between the slow learners and the fast learners from the water maze learning task in rats. Further, the fast learners show decreased *Ndfip1* mRNA and protein expression levels than the slow learners. Spatial training similarly decreases the *Ndfip1* mRNA and protein expression levels. Conversely, the *Ndfip1* conditional heterozygous (cHet) mice show enhanced spatial memory performance compared to the *Ndfip1*^*flox/WT*^ control mice. Result from co-immunoprecipitation experiment indicates that spatial training decreases the association between Ndfip1 and the E3 ubiquitin ligase Nedd4 (Nedd4-1), and we have shown that both Beclin 1 and PTEN are endogenous ubiquitination targets of Nedd4 in the hippocampus. Further, spatial training decreases endogenous Beclin 1 and PTEN ubiquitination, and increases Beclin 1 and PTEN expression in the hippocampus. On the other hand, the *Becn1* conditional knockout (cKO) mice and the *Pten* cKO mice both show impaired spatial learning and memory performance. Moreover, the expression level of Beclin 1 and PTEN is higher in the *Ndfip1* cHet mice compared with the *Ndfip1*^*flox/WT*^ control mice. Here, we have identified Ndfip1 as a candidate novel negative regulation for spatial memory formation and this is associated with increased ubiquitination of Beclin 1 and PTEN in the hippocampus.

## Introduction

It is well documented that long-term memory formation requires *de novo* RNA and protein synthesis. Previous studies have shown that inhibition of mRNA and protein synthesis impairs long-term memory formation in rats [1,2]. These results suggest that gene expression in neurons associated with learning plays an important role in memory formation. New protein synthesis and proper translational control were also demonstrated to be necessary for both long-lasting synaptic plasticity and long-term memory formation [3]. In addition, learning was shown to allow the activated synapses to recruit newly synthesized glutamate α-amino-3-hydroxy-5-methyl-4-isoxazolepropionic acid (AMPA) receptors in the hippocampus [4]. Different genes are involved in different forms of learning and memory and the pleiotropic effect may exist with disruption of a given gene [5]. Even for similar types of learning, gene expression could be different depending upon the animal species studied. Therefore, various strategies have been adopted to address the issue of gene expression associated with long-term memory formation in the past. For example, Castellucci et al. have identified several genes that are associated with long-term sensitization of gill-withdrawal reflex in *Aplysia* using two-dimensional gel analysis [6]. In a mammalian study using microarray analysis, 140 candidate genes from the rat hippocampus have been identified that are associated with water maze learning [7]. Moreover, we have previously identified the integrin-associated protein (*IAP*) gene that is associated with memory formation of inhibitory avoidance learning in rats, and this was performed by using the differential display-polymerase chain reaction (DD-PCR) strategy [8]. Later on, by using the same method, we have identified 98 cDNA fragments from the dorsal hippocampus that are differentially expressed between the fast-learning rats and slow-learning rats associated with water maze learning, and one of these cDNA fragments encodes the serum- and glucocorticoid-inducible kinase (*sgk*) gene [9]. We further demonstrate that SGK facilitates both spatial memory formation and long-term potentiation (LTP) in rats [10,11]. In addition to the *sgk* gene, we also identified another gene named the protein inhibitor of activated STAT1 (*pias1*) gene. The expression level of PIAS1 is also higher in fast learners than slow learners, and knockdown of endogenous PIAS1 expression impairs spatial memory formation [12].

In addition to protein synthesis, protein degradation has been shown to also play a role in memory consolidation [13–15] and the ubiquitin-proteasome system is an important mechanism mediating protein degradation involved in memory processing [16]. Other than the *sgk* gene and *pias1* gene reported above, we also identified other cDNA fragments that are associated with spatial memory formation in our earlier study [9]. One of these cDNA fragments encodes the rat Nedd4 (neural-precursor-cell-expressed developmentally down-regulated 4) family interacting protein 1 (*Ndfip1*) gene. Ndfip1 is an adaptor protein for the Nedd4 family of E3 ubiquitin ligases, including Nedd4, and that Ndfip1 and Nedd4 together identify target proteins for ubiquitination [17]. Ndfip1 is also necessary for the secretion of Nedd4 family proteins [18]. Ndfip1 is involved in various cellular functions. In the central nervous system, Ndfip1 was shown to regulate divalent metal transporter 1 (DMT1) and iron homeostasis and prevents metal toxicity [19,20]. Further, Ndfip1 is required for the development of neuronal dendrites and spines and it is associated with neuronal survival [21,22]. But the role of Ndfip1 in mammalian learning and memory function is not known. Here, we examined the role and molecular mechanism of Ndfip1 in spatial learning and memory formation.

The class III PI3-kinase (PI3K-III) complex is suggested to involve in a few cellular processes and Beclin 1 is believed to form the core of the PI3K-III complex for recruitment of regulatory proteins [23]. In addition, Beclin 1 was found as a polyubiquitination target of Nedd4 and Nedd4 regulates Beclin 1 stability through ubiquitination modification [24]. Further, inhibitory avoidance learning was shown to increase the level of Beclin 1 [25]. In this study, we wished to examine whether Beclin 1 is an endogenous ubiquitination target of Nedd4 involved in spatial learning and memory formation and whether Beclin 1 expression is regulated by Ndfip1.

On the other hand, phosphatase and tensin homology deleted on chromosome 10 (PTEN) is a tumor suppressor and a polyubiquitination target of Nedd4, and Nedd4-mediated PTEN ubiquitination results in PTEN proteasomal degradation [26]. Further, both LTP and long-term depression are dysregulated in Nse-Cre *Pten* cKO mice with deletion of *Pten* in dentate granule neurons [27], and that contextual fear memory is impaired in PTENα (an isoform of PTEN)-deficient mice [28]. Thus, we also wished to examine whether PTEN is an endogenous ubiquitination target of Nedd4 associated with spatial learning and memory formation and whether PTEN expression is regulated by Ndfip1.

## Materials and methods

### Differential display PCR (DD-PCR)

DD-PCR was performed in a previous study and the cDNA fragment examined here was also obtained previously [9]. Briefly, 80 arbitrary random primers (H-AP1BH-AP80, RNAimage Kit) were purchased from GenHunter (Nashville, TN). The reverse transcribed (RT) products of dorsal hippocampal tissues from three fast learners and slow learners were subjected to different amplification reactions by using these primers according to the procedures described previously [9]. Differentially expressed cDNA fragments were resolved from the sequencing gels and cloned into the PCR 2.1 TA vector (Invitrogen, Carlsbad, CA).

### Animals

Adult male Sprague-Dawley rats (250–350 g) were purchased from the BioLASCO, Taiwan. The *Ndfip1*^*flox/WT*^ mice and *Ndfip1*^*flox/flox*^ mice were generated from targeted ES cell with replaced Exon 3 of *Ndfip1* with the same exon flanked by loxP sites, but the majority of mice we obtained are the *Ndfip1*^*flox/WT*^ mice. The *Beclin 1*^*flox/flox*^ mice (strain name: *Becn1*tm1.1Yue/J, stock number: 028794) and *Pten* ^*flox/flox*^ mice (strain name: B6.129S4-*Pten*tm1Hwu/J, stock number: 006440) were purchased from Jackson Laboratory (Bar Harbor, ME). Animals were mated, bred and maintained on a 12/12 h light/dark cycle (light on at 8:00 am) at the Animal Facility of the Institute of Biomedical Sciences (IBMS), Academia Sinica with food and water continuously available. Experimental procedures follow the Guidelines of Animal Use and Care of the National Institute of Health and were approved by the Animal Committee of IBMS, Academia Sinica.

### Generation of Ndfip1^flox/WT^ conditional heterozygous (cHet) mice

*Ndfip1* knockout-first embryonic stem (ES) cell was obtained from the European Mouse Mutant Cell Repository (EuMMCR, clone number: HEPD0764_3_A03). The recombinant allele contains a FRT-hBactP-Neo-FRT-loxP cassette and a loxP site upstream and downstream of Exon 3 *Ndfip1*, respectively. Targeted ES cells were injected into blastocysts to generate chimeric mice from Transgenic Core Facility of Academia Sinica. The chimeric mice were identified by genotyping PCR using primer *Ndfip1*-F1, *Ndfip1*-F2 and *Ndfip1*-R to detect the loxP site upstream of Exon 3 and using primers Neo-F and *Ndfip1*-R to detect the FRT-flanked selection cassette. These primer sequences are shown in Table 1. For deletion the FRT-hBactP-Neo-FRT selection cassette upstream Exon 3 of *Ndfip1*, male chimeric mice containing FRT-hBactP-Neo-FRT-loxP cassette were mated with female Act-Flpe mice (stock number: 003800, Jackson lab) to generate *Ndfip1*^*flox/WT*^ mice, which were backcrossed with wild-type C57BL/6 mice for at least eight generations and confirmed by genotyping PCR using *Ndfip1*-F1, *Ndfip1*-F2, and *Ndfip1*-R primers. After eight generations, male and female *Ndfip1*^*flox/WT*^ mice were inbred to generate the *Ndfip1*^*flox/WT*^ mice and *Ndfip1*^*flox/flox*^ mice. However, most of the animals we obtained from inbreeding are the *Ndfip1*^*flox/WT*^ mice and we have therefore used this genotype of mice for the present study.

**Table 1:** List of all the primers used.

**Primer**	**Primer Sequences (5’-3’)**
*Ndfip1*-Forward-1	CTTACTTGCTGCACCATCTGGCCAG
*Ndfip1*-Forward-2	GTCGAGATATCTAGACCCAGCTTTC
Neo-Forward	AGCGAGCACGTACTCGGATG
*Ndfip1*-Reverse	CAAGCTCTAGTCCAGCTTAGGCAAC
*Nedd4-1* cDNA-Forward	ATGCAAGCTTGCCACCATGAGCTCGGACATGGCAGCC
*Nedd4-1* cDNA-Reverse	ATGCGCGGCCGCCATCAACCCCATCAAAGCCCTG
*Pten* cDNA-Forward	ATCGGAATTCATGACAGCCATCATCAAA
*Pten* cDNA-Reverse	ATCGAAGCTTTCAGACTTTTGTAATTTG
Human *ubiquitin* cDNA-Forward	ATCGCCATGGATGCAGATCTTCGTGAAGAC
Human *ubiquitin* cDNA-Reverse	ATCGGGATCCTTAGACACCCCCCCTCAAGC
*Ndfip1* siRNA-1 sense	UUUGGUCUCUCUCUAAUUAtt
*Ndfip1* siRNA-1 antisense	UAAUUAGAGAGAGACCAAAtt
*Ndfip1* siRNA-2 sense	GCAUUCCUCUUUAACUGGAtt
*Ndfip1* siRNA-2 antisense	UCCAGUUAAAGAGGAAUGCtt
*Nedd4* siRNA sense	UCAAUCGCCAUCUGAAGUUUAUCCtt
*Nedd4* siRNA antisense	AGUUAGCGGUAGACUUCAAAUAGGtt
Cre vector-Forward	ATCGGAATTCCCAAAGAAGAAGAGAAAGGTTATGTCCAATTTACTGACC
Cre vector-Reverse	ATCGGCGGCCGCCTAATCGCCATCTTCCAG
GFP vector-Forward	ATCGAGTACTGCCACCATGGAGATCGAGTGCCGCATC
GFP vector-Reverse	ATCGGGTACCGGCGAAGGCGATGGGGGTC
*Ndfip1*-Q-PCR-Forward	GCTACAACACTGCCCAGCTA
*Ndfip1*-Q-PCR-Reverse	ACCCAATCCAGTTGAAGAGG
*HRPT*-Q-PCR-Forward	GCCGACCGGTTCTGTCAT
*HPRT*-Q-PCR-Reverse	TCATAACCTGGTTCATCATCACTAATC

### Genotyping

With the primers used above, the *Ndfip1*^*+/+*^ wild-type mice have a PCR product of 305 bp in length, the *Ndfip1*^*flox/*WT^ mice have PCR products of 305 bp and 180 bp in length, and the *Ndfip1*^*flox/flox*^ mice have a PCR product of 180 bp in length. The parameters used for PCR are: 95° C for 30 s, 60° C for 30 s, 72° C for 10 s for 36 cycles, followed by a final elongation at 72° C for 90 s. The PCR product was analyzed on a 2% agarose gel.

### Drugs and drug infusion to the mouse hippocampus

N-methyl-D-aspartate (NMDA) was purchased from Tocris Bioscience (St. Louis, MO, USA) and was dissolved in PBS in a concentration of 8 mM immediately before injection. SP600125 was purchased from Sigma-Aldrich (St. Louis, MO) and was first dissolved in 100% DMSO (Sigma-Aldrich) and further diluted with PBS to a final concentration of 1 μg/μl in 45% DMSO. SP600125 was prepared immediately before injection. A volume of 0.2 μl was injected to each side of the mouse CA1 area. The injection rate was 0.1 μl/min.

### Plasmid DNA construction

Different plasmids (with different tags) were constructed as that described previously [29]. Briefly, for construction of the V5-tagged *Nedd4-1* plasmid, full-length *Nedd4-1* was cloned by amplifying the rat hippocampal *Nedd4-1* cDNA (accession # NM_012986.1) with forward and reverse primers shown in Table 1. The PCR product was sub-cloned between the *HindIII* and *NotI* sites of the mammalian expression vector pcDNA3.1-V5 vector. For construction of the Flag-tagged *PTEN* plasmid, full-length *Pten* was cloned by amplifying the rat hippocampal *Pten* cDNA (accession # NM_031606.1) with forward and reverse primers shown in Table 1. The PCR product was sub-cloned between the *EcoRI* and *HindIII* sites of the mammalian expression vector pCMVTag2B vector. The Flag-*Beclin 1* (Product ID: OHu13549D) and Flag-*Ndfip1* (Product ID: OHu20100D) plasmids were purchased from GenScript (Piscataway, NJ). For construction of the His-tagged *Ubiquitin* plasmid, full-length *ubiquitin* was cloned by amplifying the human *ubiquitin* cDNA (accession # NM_021009.7) according to that of Liu et al. [30] with forward and reverse primers shown in Table 1. The PCR product was sub-cloned between the *NcoI* and *BamHI* sites of the mammalian expression vector CMV-3 × His tag vector.

### Plasmid DNA and small interference RNA (siRNA) transfection

Different plasmids (with different tags) and *Ndfip1* siRNA (or control siRNA) were transfected to HEK293T cells for determination of Beclin 1 and PTEN ubiquitination. HEK293T cells were maintained in Dulbecco’s modified Eagle’s medium containing 10% fetal bovine serum and incubated at 37°C in a humidified atmosphere with 5% CO_2_. Two sets of *Ndfip1* siRNA were used in HEK293T cells and sequences for the sense and antisense strands are shown in Table 1. The Negative Control siRNA was used as the control. They were all synthesized from MDBio, Inc (Taipei, Taiwan). Duplex siRNA was diluted with DEPC-treated water to 50 μM, and two duplex siRNAs were used in combined *Ndfip1* siRNA-1 and *Ndfip1* siRNA-2 (equal amount). Different amounts of plasmid DNA and *Ndfip1* siRNA (100 pmol) transfections were made by using the Lipofectamine 2000 reagent (Invitrogen, Carlsbad, CA) in 6-well culture plates according to the manufacturer’s protocols. Immunoprecipitation (IP) and western blot were conducted 48 h after plasmid DNA or *Ndfip1* siRNA transfection. In addition, *Nedd4* siRNA (10 pmol) or control siRNA was and transfected to the rat CA1 area for examination of endogenous Beclin 1 ubiquitination and PTEN ubiquitination 48 h later. Transient siRNA transfection was conducted using the non-viral transfection agent polyethyleneimine (PEI) (0.1 mM) and we have previously demonstrated that PEI does not produce toxicity to hippocampal neurons [31]. The sequences for *Nedd4* siRNA sense and antisense strands are shown in Table 1.

### Lenti-NLS-Cre construction and injection to the mouse hippocampus

The lenti-NLS-Cre vector was prepared based on the construct we already have as described previously [29]. For construction of GFP-2A-NLS-Cre lentiviral vector, full-length Cre recombinase cDNA was added with nuclear localization signal (NLS) using PCR-amplification and cloned into pLenti-Tri-cistronic (ABM, Richmond, BC, Canada) to obtain a bicistronic vector expressing both GFP and NLS-Cre. The primer sequences used for the Cre vector are shown in Table 1. The PCR product was subcloned between the *EcoRI* and *NotI* sites of the lentiviral vector pLenti-Tri-cistronic (ABM). The GFP construct was cloned by amplifying the *GFP* gene from pLenti-CMV-GFP-2A-Puro-Blank (ABM) and subcloned into the pLenti-Tri-cistronic vector between *ScaI* and *KpnI* sites, upstream of the 2A peptide (a self-processing viral peptide bridge) and NLS-Cre sequences. The primer sequences used for the GFP vector are shown in Table 1. For lentivirus packaging, HEK293LTV cells (Cell Biolabs, San Diego, CA) were transfected with 1.5 μg of psPAX2 (Addgene plasmid #12260), 0.5 μg of pMD2.G (Addgene plasmid #12259), and 2 μg of pLenti-GFP-2A-NLS-Cre (or 2 μg of pLenti-CMV-GFP-2A-Puro-Blank (ABM) coding for GFP as control) using 10 μl of Lipofectamine 2000 (Invitrogen) in 6-well cell culture dishes. Lentiviral particles were collected using the speedy lentivirus purification solution (ABM) according to the manufacturer’s protocols. Cell culture medium containing lentiviral particles can be harvested for two to three times at 12 h interval until 36 h after transfection, and it was kept at 4°C for the collecting period. The collected culture medium was further clarified by centrifugation at 2,500 x g for 10 min and filtrated through a 0.45 μm syringe filter. The speedy lentivirus purification solution (ABM) was added into filtrated supernatant (1:9, v/v) containing lentiviral particles and mixed thoroughly by inversion. The lentiviral supernatant was centrifuged at 5,000 x g at 4°C for 10 min. Supernatant was then discarded and the viral pellet was re-suspended in ice cold PBS. After titration, the viral stock was stored at -80°C in aliquots. The lentivirus titer was determined by lentivirus qPCR Titer Kit (ABM) according to the manufacturer’s protocols. The final concentration of the lentiviral vector used for injection to the hippocampus is 5 x 10^8^ IU/ml.

For lentiviral vector injection, mice were anesthetized with pentobarbital (50 mg/kg, i.p.) and subjected to stereotaxic surgery without cannulation. Lentiviral vector was directly injected to their CA1 area bilaterally at the following coordinates: -1.8 mm posterior to the bregma, ±1.3 mm lateral to the midline, and -2.1 mm ventral to the skull surface. A volume of 0.2 μl was injected to each side of the CA1 area. The infusion rate was 0.1 μl/min. Spatial learning started two weeks after the lentiviral vector injection.

### Quantitative real-time PCR (Q-PCR)

Total RNA from CA1 tissue was isolated by using the RNAspin mini kit (GE Healthcare). The cDNA was generated from total RNA with Superscript III reverse transcriptase (Invitrogen). Real-time PCR analysis was performed by using the ABI PRISM 7500 real-time PCR system with *Power* SYBR Green PCR Master Mix according to the instruction manual (Applied Biosystems, Foster City, CA). The forward and reverse primer sequences used for *Ndfip1* are shown in Table 1. The primer sequences used for *HRPT* are also shown in Table 1. The cycle threshold (*Ct*) value and data were analyzed by using the 7500 system Sequence Detection Software (Applied Biosystems). Quantitative analysis of *Ndfip1* gene expression was normalized to that of *HPRT* gene expression.

### Immunoprecipitation and western blot

Immunoprecipitation (IP) and western blot for hippocampal tissue lysate were conducted according to that described previously [32]. Briefly, cell lysate, mice and rat CA1 tissue were lysed by brief sonication in RIPA lysis buffer containing 50 mM Tris-HCl (pH 7.4), 150 mM NaCl, 2 mM EDTA, 1% IGEPAL CA-630 and 20 mM N-ethylmaleimide (Catalog No. E3876-5G, Sigma-Aldrich). One tablet of protease inhibitor cocktail (Catalog No. 05892791001, cOmplete ULTRA Tablets, Mini, EDTA-free, EASYpack, Roche, Mannheim, Germany) and one tablet of phosphatase inhibitor (Catalog No. 04906837001, PhosSTOP, Roche) were added to each 10 ml of the RIPA lysis buffer. For IP of Flag-PTEN and Flag-Beclin 1, the clarified lysate (0.5 mg) was immunoprecipitated with 3 μl of anti-Flag M2 antibody (Catalog No. F1804, Sigma-Aldrich, St. Louis, MO) at 4°C for overnight. The protein G magnetic beads (30 μl, 50% slurry, GE Healthcare, Chicago, IL) were added to the IP reaction product to catch the immune complex at 4°C for 3 h. For IP of Ndfip1, the clarified lysate (0.5 mg) was immunoprecipitated with 6 μl of anti-Ndfip1 antibody (Catalog No. Ab236892, Abcam, Cambridge, UK) at 4°C for overnight. The protein A magnetic beads (60 μl, 50% slurry, GE Healthcare, Chicago, IL) were added to the IP reaction product to catch the immune complex at 4°C for 3 h. The immune complex on beads were washed three times with washing buffer containing 20 mM HEPES (pH 7.4), 150 mM NaCl, 1 mM EDTA, 1% IGEPAL CA-630, 1 mM DTT, 50 mM β-glycerophosphate, 50 mM NaF, 10 mg/ml PMSF, 4 μg/ml aprotinin, 4 μg/ml leupeptin and 4 μg/ml pepstatin and were subjected to 8% or 12% SDS-PAGE followed by transferring onto the PVDF membrane (Catalog No. IPVH00010, Millipore, Bedford, MA). Western blot was conducted using the following antibodies: anti-Ndfip1 (1:3000; Catalog No. Ab236892, Abcam, Cambridge, UK), anti-Nedd4 (1:2000; Catalog No. 5344S, Cell Signaling, Danvers, MA), anti-Beclin 1 (1:2000; Catalog No. 3738S, Cell Signaling), anti-PTEN (1:2000; Catalog No. 9188S, Cell Signaling), anti-ubiquitin (1:3000, Catalog No. 3936, Cell Signaling), anti-His (1:4000, Catalog No. 66005-Ig, Proteintech, Rosemont, IL), anti-Flag M2 (1:8000; Sigma-Aldrich), anti-V5 (1:8000; Catalog No. MCA2895, AbD Serotec, Kidlington, UK) and anti-actin (1:200000; Millipore; Catalog No. MAB1501) antibodies. The secondary antibody used was HRP-conjugated goat-anti rabbit IgG antibody or HRP-conjugated goat-anti mouse IgG antibody (1:8000, Catalog No. 111-035-003 and 115-035-003, Jackson ImmunoResearch, West Grove, PA). The secondary antibody used for co-IP experiment was HRP-conjugated goat-anti rabbit IgG light chain antibody (1:6000, Catalog No. 112-035-175, Jackson ImmunoResearch) or HRP-conjugated goat-anti mouse IgG light chain antibody (1:6000, Catalog No. 115-035-174, Jackson ImmunoResearch). Membrane was developed by reacting with chemiluminescence HRP substrate (Millipore) and was exposed to the LAS-3000 image system (Fujifilm, Tokyo, Japan) for visualization of protein bands. The protein bands were quantified by using the NIH Image J Software.

### Immunohistochemistry

For immunohistochemical staining of GFP in the CA1 area of the mouse brain, mice were anesthetized with pentobarbital (50 mg/kg, i.p.) and perfused with ice-cold PBS, followed by 4% paraformaldehyde. Brains were removed and post-fixed in 20% sucrose/4% paraformaldehyde solution for 20–48 h. Brains were then frozen, cut into 30-μm sections on a cryostat and mounted on gelatin-coated slides. Brain sections were rinsed with 1 X PBS for 10 min and permeabilized with pre-cold EtOH/CH3COOH (95%:5%) for 10 min, followed by 1 X PBS for 10 min for three times. The sections were pre-incubated in a blocking solution containing 3% bovine serum albumin (BSA) and 0.1% Triton X-100 in 1 X TBS for 1 h, followed by 1 X PBS for 10 min for three times. For examination of lentiviral vector transduction, brain sections containing the CA1 area were prepared for visualization of GFP (green) fluorescence. For immunofluorescence detection of the nucleus, tissue sections were added with 20 μl of the Fluoromount-G mounting medium with DAPI (SouthernBiotech, Birmingham, AL). Photomicrographs were taken using a Zeiss LSM700 confocal microscope.

### Water maze learning

For spatial acquisition adopted in the present study, the water maze used was a plastic, circular pool, 1.2 m in diameter and 25 cm in height that was filled with water (25 ± 2°C) to a depth of 16 cm. A circular platform of 10 cm in diameter was placed at a specific location away from the edge of the pool. The top of the platform was submerged 0.6 cm below the water surface. Water was made cloudy by adding milk powder. Distinctive, visual cues were set on the wall. For spatial acquisition, animals were subjected to three trials a day (as one session) with one given early in the morning, one given in the early afternoon and the other one given in the late afternoon. The acquisition procedure lasted for 5 days (for 5 sessions) and a total of 15 trials were given. For these trials, animals were placed at different starting positions spaced equally around the perimeter of the pool in a random order. Animals were given 60 sec to find the platform. If an animal could not find the platform within 60 sec, it was guided to the platform and was allowed to stay on the platform for 20 sec. The time that each animal took to reach the platform was recorded as the escape latency. A probe trial of 60 sec was given on day 6 to test their memory retention. Animals were placed in the pool with the platform removed and the time they spent in each quadrant (target quadrant, left quadrant, opposite quadrant and right quadrant), the total distance travelled in the target quadrant and their swim speed were recorded. For spatial training, the procedures were the same as that for spatial acquisition except that training lasted for two consecutive days (for *Ndfip1* mRNA measure) or three consecutive days (for Ndfip1 protein measure). Animals were sacrificed at the end of the last training trial.

For screening of the fast-learning rats and slow-learning rats, the same criteria used in a previous study were adopted here [9]. Briefly, animals that reached the escape latency smaller than 30 sec by the end of the third training session were designated as the fast-learning rats (fast learners). Animals that did not reach this escape latency until the end of the seventh training session were designated as the slow-learning rats (slow learners).

For the visible platform learning experiment, a flag was mounted on the platform and the platform was 2.5 cm above the water surface so the animals can visualize the flag and indentify the location of the platform. In addition, milk powder was not added to the swimming pool to make it cloudy.

### Statistical analysis

Spatial acquisition data were analyzed with two-way analysis of variance (ANOVA) with repeated measure followed by post-hoc Newman-Keuls multiple comparisons (represented by q value). Probe trial performance (time spent in the target quadrant and distance travelled in the target quadrant) and all biochemical data were analyzed with the Student’s t-test. Values of *p* < 0.05 were considered significant (* *p* < 0.05, ** *p* < 0.01, *** *p* ≦ 0.001).

## Results

### Identification of the Nedd4 family interacting protein 1 (*Ndfip1*) gene from DD-PCR

**Fig 1:**
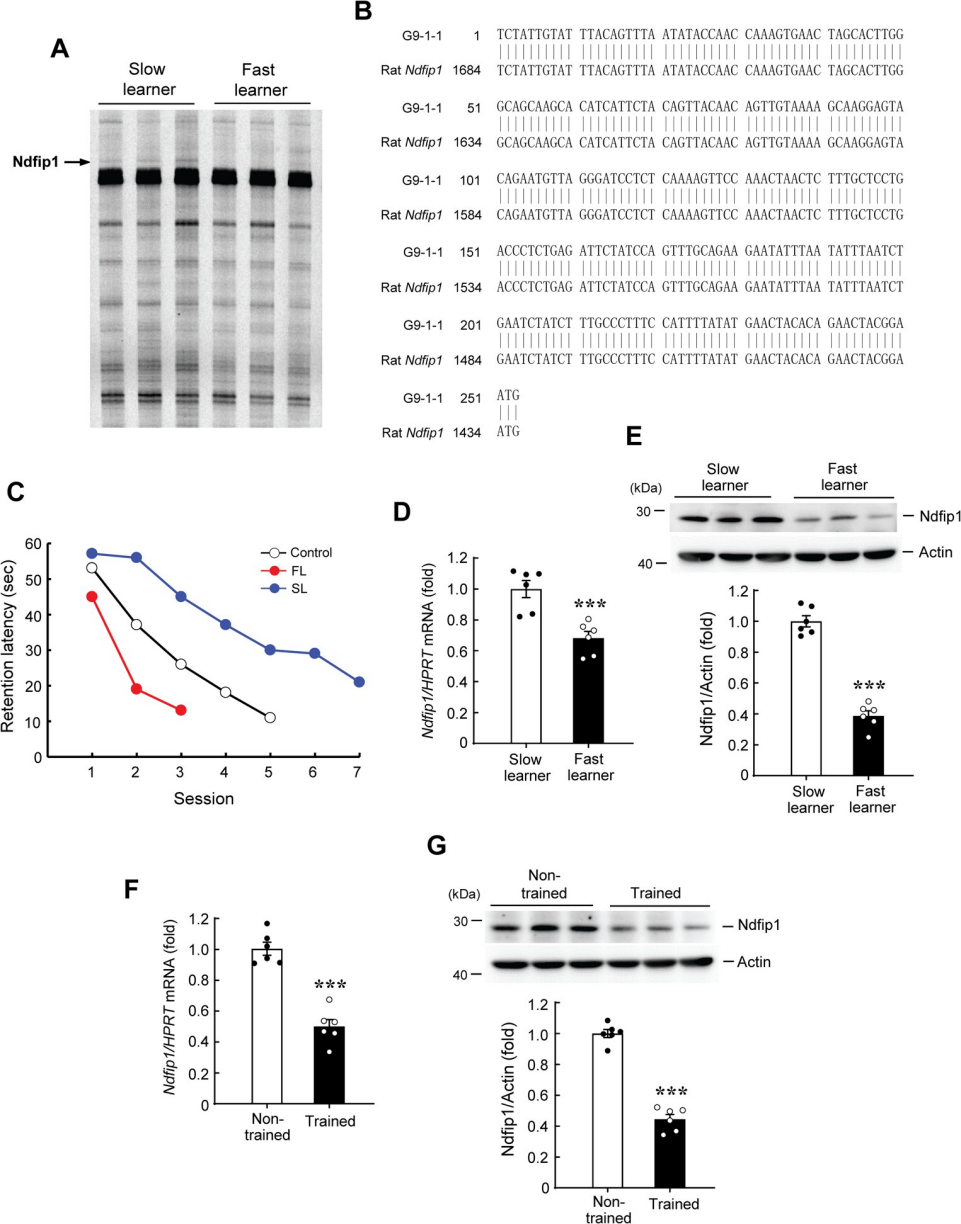
Identification of the *Ndfip1* gene and Ndfip1 expression is decreased by spatial training. **(A)** DD-PCR of hippocampal RNA associated with water maze learning in rats. One cDNA fragment (indicated by the arrow) was differentially expressed between the fast learners and the slow learners. **(B)** Alignment of the sequence of G9-1-1 (the arbitrary primers used) with rat *Ndfip1*. The numbers correspond to the *Ndfip1* cDNA sequences. Vertical lines indicate identity and 100% sequence homology was found between these two. **(C)** Acquisition performance of fast learners (N = 6), slow learners (N = 6), and control rats (N = 22) from the water maze learning task. FL: Fast learners; SL: Slow learners. Data are expressed as mean values. **(D)** *Ndfip1* mRNA level in the CA1 area is lower in fast learners than slow learners (t_1,10_ = 4.57, *p* = 0.001). **(E)** Ndfip1 protein expression in the CA1 area is lower in fast learners than slow learners (t_1,10_ = 12.43, *p* < 0.001). **(F)** *Ndfip1* mRNA level in the CA1 area is lower in trained rats than the non-trained (swimming control) rats (t_1,10_ = 8.15, *p* < 0.001). **(G)** Ndfip1 protein expression in the CA1 area is lower in trained rats than the non-trained rats (t_1,10_ = 13.79, *p* < 0.001). N = 6 each group. Data are expressed as individual values and mean ± SEM. # *p*≦0.001.

By using DD-PCR we have previously identified 98 cDNA fragments that are differentially expressed in the dorsal hippocampus between fast learners and slow learners from the water maze learning task [9]. When a specific primer set H-AP53 (5’-end primer sequence as 5’-AAGCTTCCTCTAT-3’) and H-T11C (3’-end primer sequence as 5’-AAGCTTTTTTTTTTTC-3’) was used, one identified cDNA fragment of 253 bp in length (designated as G9-1-1) showed 100% sequence homology to the 3’-end region of the rat *Ndfip1* gene (data accession number for *Ndfip1*: NM_001013059.1) (Fig 1A and 1B). This gene was categorized as one of the unknown genes in our previous study [9]. The expression level of this gene is lower in the fast learners than slow learners (Fig 1A).

### Spatial training decreases Ndfip1 expression in the rat hippocampus

To examine the role of Ndfip1 in spatial learning, we first screened another batch of rats and obtained a separate group of fast learners and slow learners using the same criteria and procedures adopted from our previous study (Fig 1C) [9]. We then examined the *Ndfip1* mRNA level in one side of the CA1 area from these fast learners and slow learners using quantitative real-time PCR (Q-PCR). The result revealed that *Ndfip1* mRNA level is higher in the slow learners than fast learners (Fig 1D). Another side of the CA1 tissue from the same animals was subjected to western blot determination of Ndfip1 protein expression. Results revealed that the Ndfip1 protein level is similarly higher in slow learners than fast learners (Fig 1E). The above results suggest that Ndfip1 expression is negatively associated with spatial acquisition. Based on these results, we expect that spatial training should decrease the expression level of Ndfip1. To test this hypothesis, a different batch of rats was randomly divided to the trained group or the non-trained group. Animals in the trained group were subjected to regular water maze learning, as described in the Method Section. Animals in the non-trained group swam for the same period of time for each trial as that of the trained animals except that the visual cues and platform were both removed. Animals were sacrificed after training and their CA1 tissue was dissected out for *Ndfip1* mRNA and protein level determination. Result from Q-PCR analysis revealed that spatial training decreased *Ndfip1* mRNA level (Fig 1F). Meanwhile, spatial training also decreased Ndfip1 protein expression level (Fig 1G). Spatial learning is known to activate the glutamate NMDA receptor. If spatial training downregulated the expression of Ndfip1, it is expected that NMDA receptor activation would yield similar result. This speculation was examined here. Rats were randomly divided to two groups and received acute intra-hippocampal PBS or NMDA (8 mM) injection. For the purpose of evaluating the effectiveness of NMDA injection, animals were sacrificed 30 min after PBS or NMDA injection and their CA1 tissue was dissected out and subjected to western blot determination of MAPK/ERK phosphorylation level. Ndfip1 expression was also determined in the same tissue lysate. Result revealed that NMDA decreased Ndfip1 expression as soon as 30 min after injection. Meanwhile, it increased the phosphorylation level of ERK1 and ERK2, but the expression level of ERK1 and ERK2 was not altered (S1 Fig).

### Spatial learning and memory is enhanced in *Ndfip1* cHet mice

**Fig 2:**
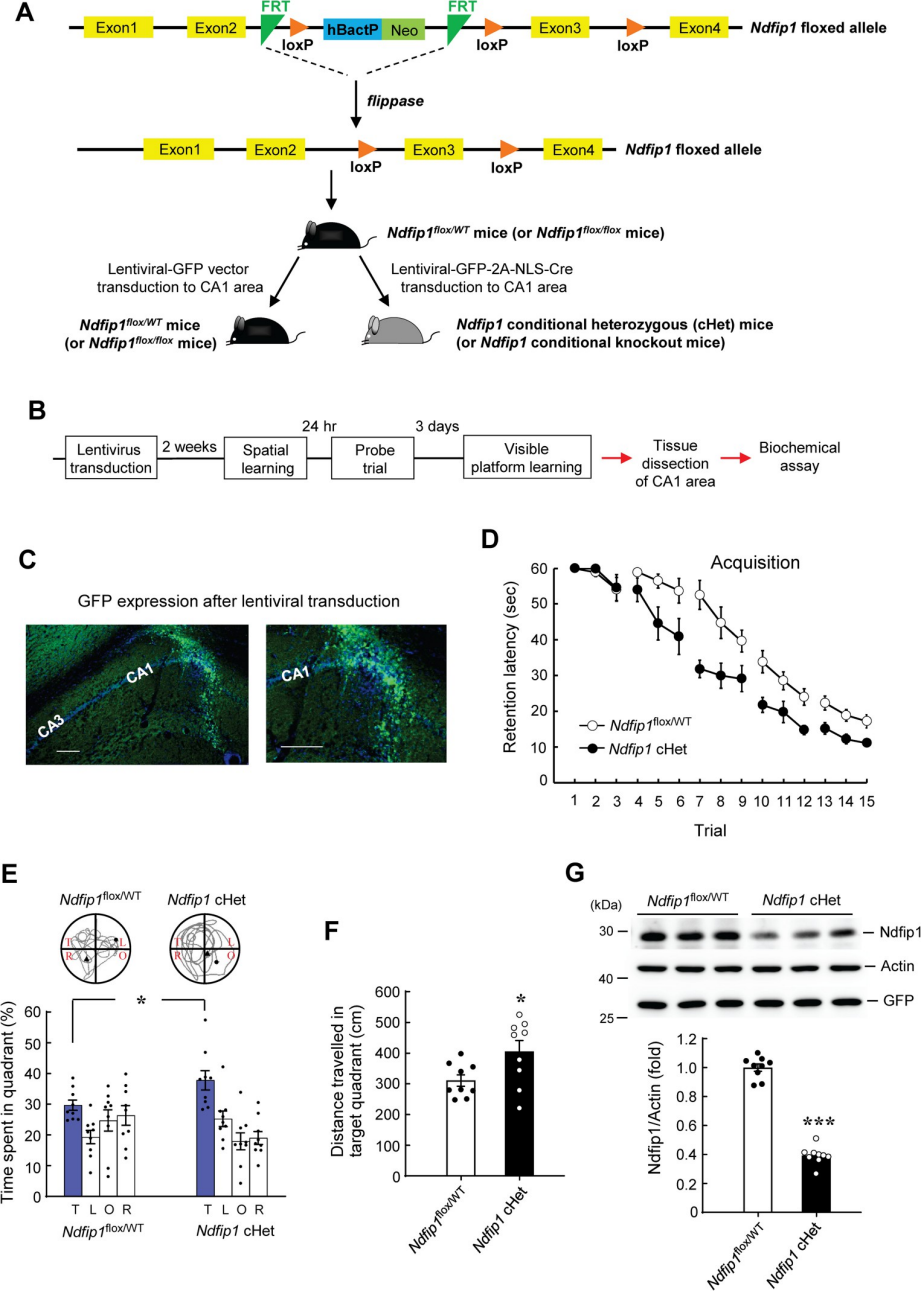
Spatial learning and memory is improved in *Ndfip1* conditional heterozygous (cHet) mice. **(A)** Schematic illustration showing the strategy of generating the *Ndfip1*^flox/WT^ mice. The inserted cassette is composed of a flippase recombination enzyme recognition target (FRT), neomycin resistance gene and Cre recombinase recognition target (loxP). The first loxP site is followed by the human β-actin promoter driving the neomycin resistance gene, a second FRT site and a second loxP site. A third loxP site is inserted downstream of the targeted Exon 3. *Ndfip1* Exon 3 is flanked by loxP sites. Mice with *Ndfip1* floxed allele were generated by crossing male chimeric mice containing FRT-neo-FRT-loxP cassette with female Act-Flp (flippase) mice. *Ndfip1*^flox/WT^ mice were inbred to obtain *Ndfip1*^flox/WT^ mice or conditional *Ndfip1* (*Ndfip1*^flox/flox^) mice. Lenti-GFP vector or lenti-GFP-2A-NLS-Cre vector was transducted to the CA1 area of *Ndfip1*^flox/WT^ mice. **(B)** Schedule of lentivirus transduction to *Ndfip1*^flox/WT^ mice, behavioral testing and tissue dissection. **(C)** Immunohistochemistry showing the location of lenti-GFP vector transduction and GFP expression (green color) in the CA1 area. DAPI staining is shown in blue color. Scale bar is 100 μm (left panel). A picture at a higher magnification is shown in the right panel. Scale bar is 100 μm. **(D)** *Ndfip1*^flox/WT^ mice that received lenti-GFP vector transduction (*Ndfip1*^flox/WT^ mice) or lenti-GFP-2A-NLS-Cre transduction (*Ndfip1* cHet mice) were subjected to water maze learning two weeks later. The *Ndfip1* cHet mice showed a better acquisition performance than the *Ndfip1*^flox/WT^ mice (F_1,16_ = 24.66, *p* < 0.001). **(E)** Probe trial performance (t_1,16_ = 2.27, *p* < 0.05 for the target quadrant) and **(F)** Distance travelled in the target quadrant for the probe trial test (t_1,16_ = 2.39, *p* < 0.05) from the same animals as that in (D). The *Ndfip1* cHet mice showed a better retention performance and travelled more in the target quadrant than the *Ndfip1*^flox/WT^ mice **(G)** The *Ndfip1* cHet mice showed a decreased Ndfip1 expression level than the *Ndfip1*^flox/WT^ mice (t_1,16_ = 18.46, *p* < 0.001). The GFP expression level is similar for these two groups of mice. N = 9 each group. Data are expressed as individual values and mean ± SEM. * *p* < 0.05 and *** *p* < 0.001.

To further examine the role of Ndfip1 in spatial learning and memory formation, we have generated the *Ndfip1*^*flox/WT*^ mice as described in the Method Section. The strategy for generating the *Ndfip1* cHet mice is shown in Fig 2A. For the purpose of obtaining the *Ndfip1* cHet mice, one group of *Ndfip1*^*flox/WT*^ mice received intra-hippocampal lenti-GFP-2A-NLS-Cre vector transduction. The other group of *Ndfip1*^*flox/WT*^ mice received intra-hippocampal lenti-GFP-vector transduction and served as the control group. All the animals were subjected to water maze learning two weeks after lentiviral vector transduction. The schedule for lentivirus transduction, behavioral testing and biochemical assays is shown in Fig 2B. The expression of GFP (green) in the mouse CA1 area after lentivirus transduction is shown in Fig 2C. Results revealed that spatial acquisition performance was significantly improved in *Ndfip1* cHet mice compared to the *Ndfip1*^*flox/WT*^ mice (Fig 2D). These animals were subjected to probe trial test the next day after the last acquisition trial. Results revealed that *Ndfip1* cHet mice spent more time in the target quadrant than that of the *Ndfip1*^*flox/WT*^ mice (Fig 2E). They also made more travelling in the target quadrant than the *Ndfip1*^*flox/WT*^ mice did (Fig 2F). But the swim speed of these two groups of mice is similar (S2A Fig).

The same animals were also subjected to visible platform learning after the probe trial test. The result showed that the visible platform performance between these two groups of mice is not different (S2B Fig). These results indicated that the visual and motor functions were not altered in *Ndfip1* cHet mice. Animals were sacrificed at the end of visible platform learning and their CA1 tissue was dissected out for determination of Ndfip1 protein expression. Result revealed that Ndfip1 expression level was significantly decreased in the *Ndfip1* cHet mice compared to the *Ndfip1*^*flox/WT*^ mice, but the GFP expression level is similar between these two groups of mice, indicating that the lentiviral vector was effectively transducted to these animals in similar amount (Fig 2G).

### Spatial training decreases the association between Ndfip1 and Nedd4 and decreases endogenous Beclin 1 ubiquitination in the hippocampus

**Fig 3:**
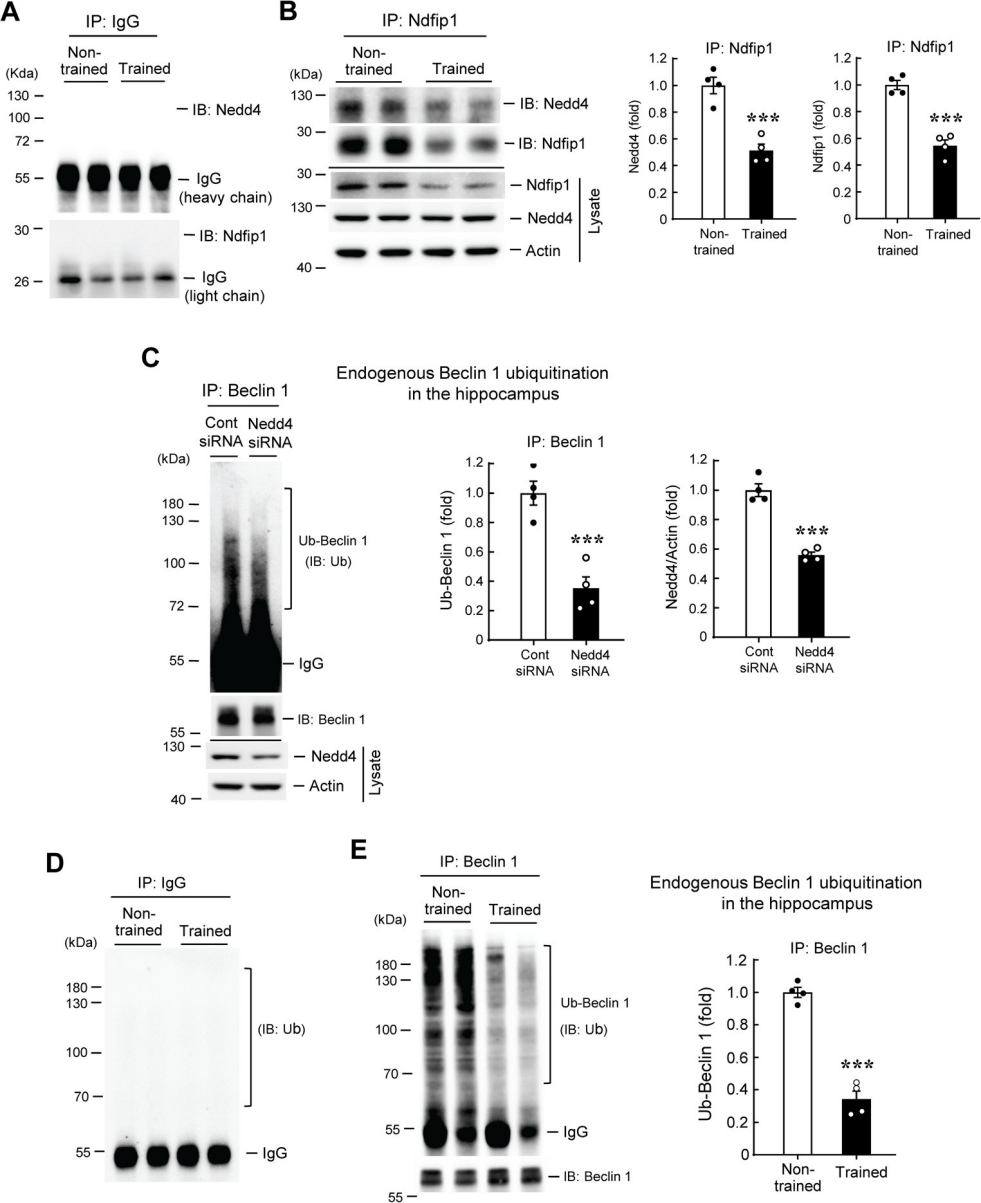
Spatial training decreases the association between Ndfip1 and Nedd4 and decreases endogenous Beclin 1 ubiquitination in the hippocampus. **(A)** The CA1 tissue lysates from non-trained and trained rats were immunoprecipitated with IgG and immunoblotted with anti-Nedd4 and anti-Ndfip1 antibodies to serve as the control group. No specific band was identified. **(B)** The CA1 tissue lysates from different non-trained and trained rats were immunoprecipitated with anti-Ndfip1 antibody and immunoblotted with anti-Nedd4 and anti-Ndfip1 antibodies. The association between Ndfip1 and Nedd4 is decreased in trained rats. The tissue lysate was also subjected to western blotting using anti-Ndfip1 and anti-Nedd4 antibodies. The Ndfip1 expression level is decreased in both the IP product and the lysate in trained rats. The quantified results from four independent experiments are shown in the right panel (t_1,6_ = 6.18 for Nedd4 expression, *p* < 0.001 and t_1,6_ = 8.23 for Ndfip1 expression, *p* < 0.001). **(C)** Control siRNA or Nedd4 siRNA (10 pmol) was transfected to the rat CA1 area. The CA1 tissue lysates from these animals were immunoprecipitated with anti-Beclin 1 antibody and immunoblotted with anti-ubiquitin and anti-Beclin 1 antibodies. Endogenous Beclin 1 ubiquitination level is decreased in Nedd4 siRNA-transfected rats (t_1,6_ = 5.78, *p* = 0.001). The same cell lysates were also subjected to western blot determination of Nedd4 expression, and Nedd4 expression level is decreased in Nedd4 siRNA-transfected rats (t_1,6_ = 9.26, *p* < 0.001). N = 4 each group. **(D)** The CA1 tissue lysates from non-trained and trained rats were immunoprecipitated with IgG and immunoblotted with anti-ubiquitin antibody to serve as the control group. No specific band was identified. **(E)** The CA1 tissue lysates from different non-trained and trained rats were immunoprecipitated with anti-Beclin 1 antibody and immunoblotted with anti-ubiquitin and anti-Beclin 1 antibodies. Endogenous Beclin 1 ubiquitination level is decreased in trained rats (t_1,6_ = 11.29, *p* < 0.001). N = 4 each group. Data are expressed as individual values and mean ± SEM. *** *p* < 0.001. Ub: Ubiquitin.

As mentioned above, Ndfip1 is an adaptor protein for Nedd4 [17], and we have found that Ndfip1 expression is decreased by spatial training; here we examined the relationship between Ndfip1 and Nedd4 associated with spatial learning. Co-IP experiment was conducted to examine this issue. We first carried out a control experiment. The CA1 tissue lysates from non-trained and trained rats were immunoprecipitated with IgG and immunoblotted with anti-Nedd4 and anti-Ndfip1 antibodies. Results showed that other than the light-chain and heavy-chain bands, no specific band was observed (Fig 3A). Next, the CA1 tissue lysates from different non-trained and trained rats were immunoprecipitated with anti-Ndfip1 antibody and immunoblotted with anti-Nedd4 and anti-Ndfip1 antibodies. Result indicated that Ndfip1 is associated with Nedd4 in the hippocampus, but this association is significantly decreased in trained animals compared with the non-trained animals. On the other hand, the Ndfip1 expression level is consistently decreased in both the IP product and the lysates from trained animals, but the Nedd4 expression level in the lysate is not altered (Fig 3B). Based on these results, we hypothesized that Ndfip1 may impair spatial memory through its interaction with Nedd4 and subsequent ubiquitination of Nedd4 target proteins, whereas these target proteins play a role in facilitating spatial memory formation.

As described above, Beclin 1 is an ubiquitination target of Nedd4 [24], here we first examined whether Ndfip1 regulates Beclin 1 ubiquitination by Nedd4. Different plasmids together with V5-Nedd4 (or V5-vector) plasmid were co-transfected to HEK293T cells. Cell lysate was immunoprecipitated with anti-Flag antibody and immunoblotted with anti-His antibody. Result revealed that enhanced Beclin 1 ubiquitination was observed when V5-Nedd4 was transfected to the cells compared with V5-vector transfection (S3A Fig). But Beclin 1 ubiquitination by Nedd4 was markedly decreased when *Ndfip1* siRNA was co-transfected to HEK293T cells compared with control siRNA transfection (S3B Fig). Next, we examined whether Beclin 1 is an endogenous ubiquitination target of Nedd4 in the hippocampus and whether endogenous Beclin 1 ubiquitination is altered by spatial training. To examine the first issue, control siRNA or Nedd4 siRNA was transfected to the rat CA1 area. The CA1 tissue lysate was immunoprecipitated with anti-Beclin 1 antibody and immunoblotted with anti-ubiquitin antibody. Results showed that endogenous Beclin 1 ubiquitination level is markedly decreased by Nedd4 siRNA transfection. Nedd4 siRNA transfection also effectively decreased Nedd4 expression (Fig 3C).

Next, we aimed to identify the role of Beclin 1 in spatial learning. We first conducted a control experiment. The CA1 tissue lysates from non-trained and trained rats were immunoprecipitated with IgG and immunoblotted with anti-ubiquitin antibody. Results showed that other than the IgG heavy-chain, no specific band was observed (Fig 3D). The CA1 tissue lysates from different non-trained and trained rats were immunoprecipitated with anti-Beclin 1 antibody and immunoblotted with anti-ubiquitin antibody. Result showed that endogenous Beclin 1 ubiquitination level is significantly decreased in trained animals compared with the non-trained controls (Fig 3E).

### Spatial training increases Beclin 1 expression and spatial learning and memory is impaired in *Becn1* cKO mice

**Fig 4:**
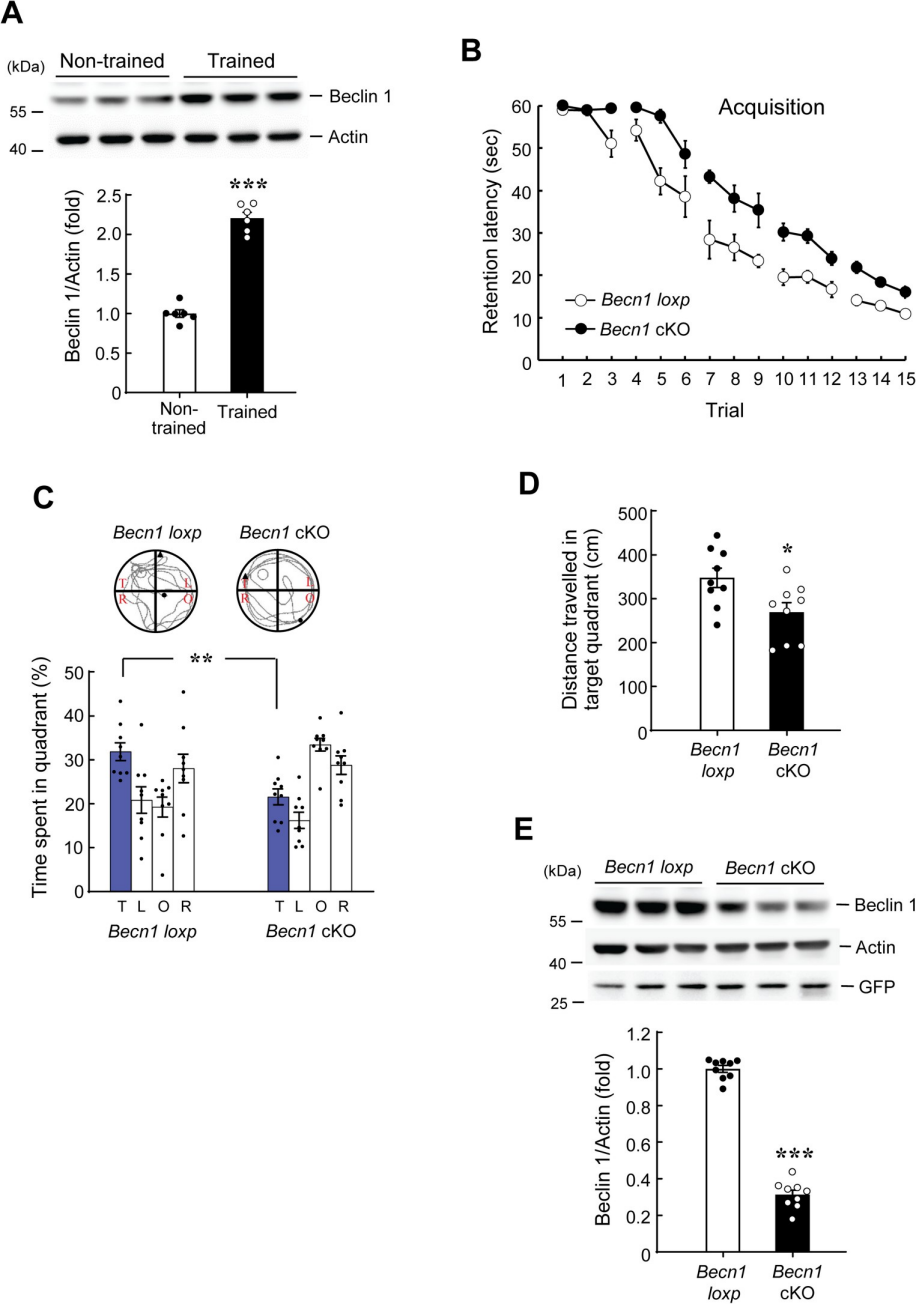
Spatial training increases Beclin 1 expression, and spatial learning and memory is impaired in *Becn1* cKO mice. **(A)** Rats were subjected to water maze training or swimming control (non-trained) and were sacrificed three days later. Their CA1 tissue was subjected to western blot determination of Beclin 1 expression (N = 6 each group, the same animals in Fig 1F). Water maze training increased Beclin 1 expression level (t_1,10_ = 11.95, *p* < 0.001). **(B)** The *Becn1 loxp* mice (that received lenti-GFP vector transduction) and *Becn1* cKO mice (that received lenti-GFP-2A-NLS-Cre vector transduction) were subjected to water maze learning two weeks after lentivirus transduction. The *Becn1* cKO mice showed impaired acquisition performance (N = 9 each group) (F_1,16_ = 33.75, *p* < 0.001). **(C)** Probe trial performance (t_1,16_ = 3.79, *p* < 0.01 for the target quadrant) and **(D)** Distance travelled in the target quadrant (t_1,16_ = 2.55, *p* < 0.05) from the same animals as that in (B). The *Becn1* cKO mice showed impaired retention performance and made less travelling in the target quadrant than the *Becn1 loxp* mice. **(E)** The Beclin 1 expression level is decreased in the *Becn1* cKO mice than the *Becn1 loxp* mice (t_1,16_ = 22.16, *p* < 0.001). The GFP expression level is similar for these two groups of mice. Data are expressed as individual values and mean ± SEM. * *p* < 0.05, ** *p* < 0.01 and *** *p* < 0.001.

Based on the result that spatial training decreases endogenous Beclin 1 ubiquitination in the hippocampus, we expect that spatial training should increase the expression level of Beclin 1 in the hippocampus. This issue was examined here. The CA1 tissue lysates from non-trained and trained animals described above (from Fig 1F) were subjected to western blot determination of Beclin 1 expression. Result revealed that Beclin 1 expression level was significantly increased by spatial training (Fig 4A). Next, we examined the role of Beclin 1 in spatial memory formation. The *Becn1*^*flox/flox*^ mice were randomly divided to two groups. One group of mice received intra-hippocampal lenti-GFP-2A-NLS-Cre transduction and the other group of mice received intra-hippocampal lenti-GFP vector transduction. They were then subjected to water maze learning. The injection and behavioral testing paradigm is the same as that described in Fig 2B. Result revealed that spatial acquisition performance was significantly impaired in *Becn1* cKO mice (Fig 4B). In addition, the *Becn1* cKO mice spent less time in the target quadrant (Fig 4C) and they also made less travelling in the target quadrant during the probe trial test (Fig 4D). But the swim speed of these two groups of mice is similar (S2C Fig). These animals were also subjected to visible platform learning after the probe trial test. Result showed that the acquisition performance between the *Becn1* cKO mice and *Becn1 loxp* mice was not different (S2D Fig). This result indicated that the visual and motor functions were not altered in *Becn1* cKO mice. Animals were sacrificed at the end of visible platform learning and their CA1 tissue was dissected out for determination of Beclin 1 expression. Western blot analysis indicated that Beclin 1 expression level is significantly decreased in *Becn1* cKO mice, but GFP expression level is similar between these two groups of mice, indicating that the lentiviral vector was effectively transducted to these animals in similar amount (Fig 4E).

### Spatial training decreases endogenous PTEN ubiquitination and *Pten* cKO mice show impaired spatial learning and memory

In this set of experiments, we examined whether spatial training alters endogenous PTEN ubiquitination and the role of PTEN in spatial memory formation. To be related to Ndfip1, we first studied whether Ndfip1 regulates Nedd4-mediated PTEN ubiquitination. Different plasmids together with V5-Nedd4 (or V5-vector) plasmid were co-transfected to HEK293T cells. Cell lysate was immunoprecipitated with anti-Flag antibody and immunoblotted with anti-His antibody. Result revealed that PTEN ubiquitination is increased when V5-Nedd4 plasmid was transfected compared with V5-vector transfection (S4A Fig). But PTEN ubiquitination by Nedd4 was markedly decreased when *Ndfip1* siRNA was co-transfected to HEK293T cells compared with control siRNA transfection (S4B Fig).

**Fig 5:**
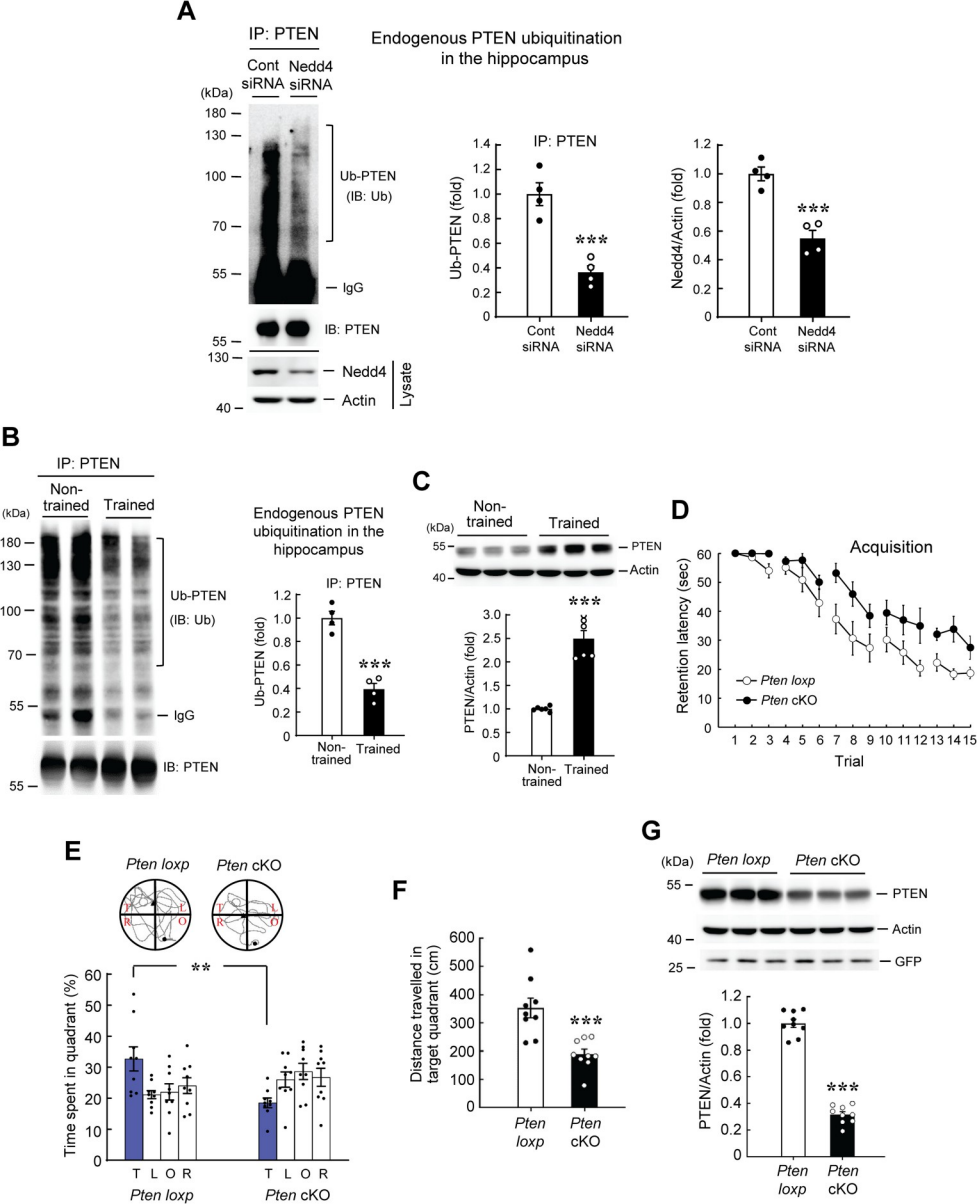
Spatial training decreases endogenous PTEN ubiquitination and *Pten* cKO mice show impaired spatial learning and memory. **(A)** Control siRNA or Nedd4 siRNA (10 pmol) was transfected to the rat CA1 area. The CA1 tissue lysates from these animals were immunoprecipitated with anti-PTEN antibody and immunoblotted with anti-ubiquitin and anti-PTEN antibodies. Endogenous PTEN ubiquitination level is decreased in Nedd4 siRNA-transfected rats (t_1,6_ = 5.92, *p* = 0.001). The same cell lysates were also subjected to western blot determination of Nedd4 expression, and Nedd4 expression level is decreased in Nedd4 siRNA-transfected rats (t_1,6_ = 6.22, *p* < 0.001). N = 4 each group. **(B)** The CA1 tissue lysates from non-trained and trained rats were immunoprecipitated with anti-PTEN antibody and immunoblotted with anti-ubiquitin and anti-PTEN antibodies. Endogenous PTEN ubiquitination level is decreased in trained rats (t_1,6_ = 7.91, *p* < 0.001). N = 4 each group. **(C)** Rats were subjected to water maze training or swimming control (non-trained) and were sacrificed three days later. Their CA1 tissue was subjected to western blot determination of PTEN expression, and the PTEN expression level is increased in trained rats (t_1,10_ = 8.59, *p* < 0.001). N = 6 each group. **(D)** The *Pten loxp* mice (that received lenti-GFP vector transduction) and *Pten* cKO mice (that received lenti-GFP-2A-NLS-Cre vector transduction) were subjected to water maze learning two weeks after lentivirus transduction. The *Pten* cKO mice showed impaired acquisition performance (N = 9 each group) (F_1,16_ = 18.07, *p* < 0.001). **(E)** Probe trial performance (t_1,16_ = 3.36, *p* < 0.01 for the target quadrant) and **(F)** Distance travelled in the target quadrant (t_1,16_ = 4.23, *p* < 0.001) from the same animals as that in (D). The *Pten* cKO mice showed impaired retention performance and made less travelling in the target quadrant than the *Pten loxp* mice. **(G)** The PTEN expression level is decreased in the *Pten* cKO mice than the *Pten loxp* mice (t_1,16_ = 17.96, *p* < 0.001). The GFP expression level is similar for these two groups of mice. Data are expressed as individual values and mean ± SEM. ** *p* < 0.01 and *** *p*≦0.001. Ub: Ubiquitin.

Next, we examined whether PTEN is an endogenous ubiquitination target of Nedd4 in the hippocampus. Control siRNA or Nedd4 siRNA was transfected to the rat CA1 area. The CA1 tissue lysate was immunoprecipitated with anti-PTEN antibody and immunoblotted with anti-ubiquitin antibody. Results showed that endogenous PTEN ubiquitination level is significantly decreased by Nedd4 siRNA transfection. Nedd4 siRNA transfection also decreased Nedd4 expression (Fig 5A). Next, we examined whether endogenous PTEN ubiquitination is associated with spatial training. The hippocampal tissue lysates from non-trained and trained rats were immunoprecipitated with anti-PTEN antibody and immunoblotted with anti-ubiquitin antibody. Result revealed that endogenous PTEN ubiquitination level is significantly decreased in trained animals compared with the non-trained controls (Fig 5B). Based on the result that spatial training decreased endogenous PTEN ubiquitination in the hippocampus, we expect that spatial training should increase the expression level of PTEN in the hippocampus. We addressed this issue here. The CA1 tissue lysates from non-trained and trained animals described above (from Fig 1F) were subjected to western blot determination of PTEN expression. Result showed that spatial training markedly increased the expression level of PTEN (Fig 5C). Lastly, we examined the role of PTEN in spatial memory formation by using the *Pten* cKO mice. The *Pten*^*flox/flox*^ mice were randomly divided to two groups. One group of mice received intra-hippocampal lenti-GFP-2A-NLS-Cre transduction and the other group of mice received intra-hippocampal lenti-GFP vector transduction. They were then subjected to water maze learning as described above. Result revealed that the spatial acquisition performance was significantly impaired in *Pten* cKO mice (Fig 5D). These animals also spent less time in the target quadrant (Fig 5E) and made less travelling in the target quadrant (Fig 5F) during the probe trial test. But the swim speed of these two groups of mice is similar (S2E Fig). These animals were also subjected to visible platform learning after the probe trial test. Result showed that the acquisition performance between the *Pten* cKO mice and *Pten loxp* mice was not different (S2F Fig). This result indicated that the visual and motor functions were not altered in *Pten* cKO mice. Animals were sacrificed at the end of visible platform learning and their CA1 tissue was dissected out for determination of PTEN expression. Western blot analysis revealed that PTEN expression level is significantly decreased in *Pten* cKO mice, but the GFP expression level is similar between these two groups of mice, indicating that the lentiviral vector was effectively transducted to these animals in similar amount (Fig 5G).

### Beclin 1 expression and PTEN expression are increased in *Ndfip1* cHet mice

**Fig 6:**
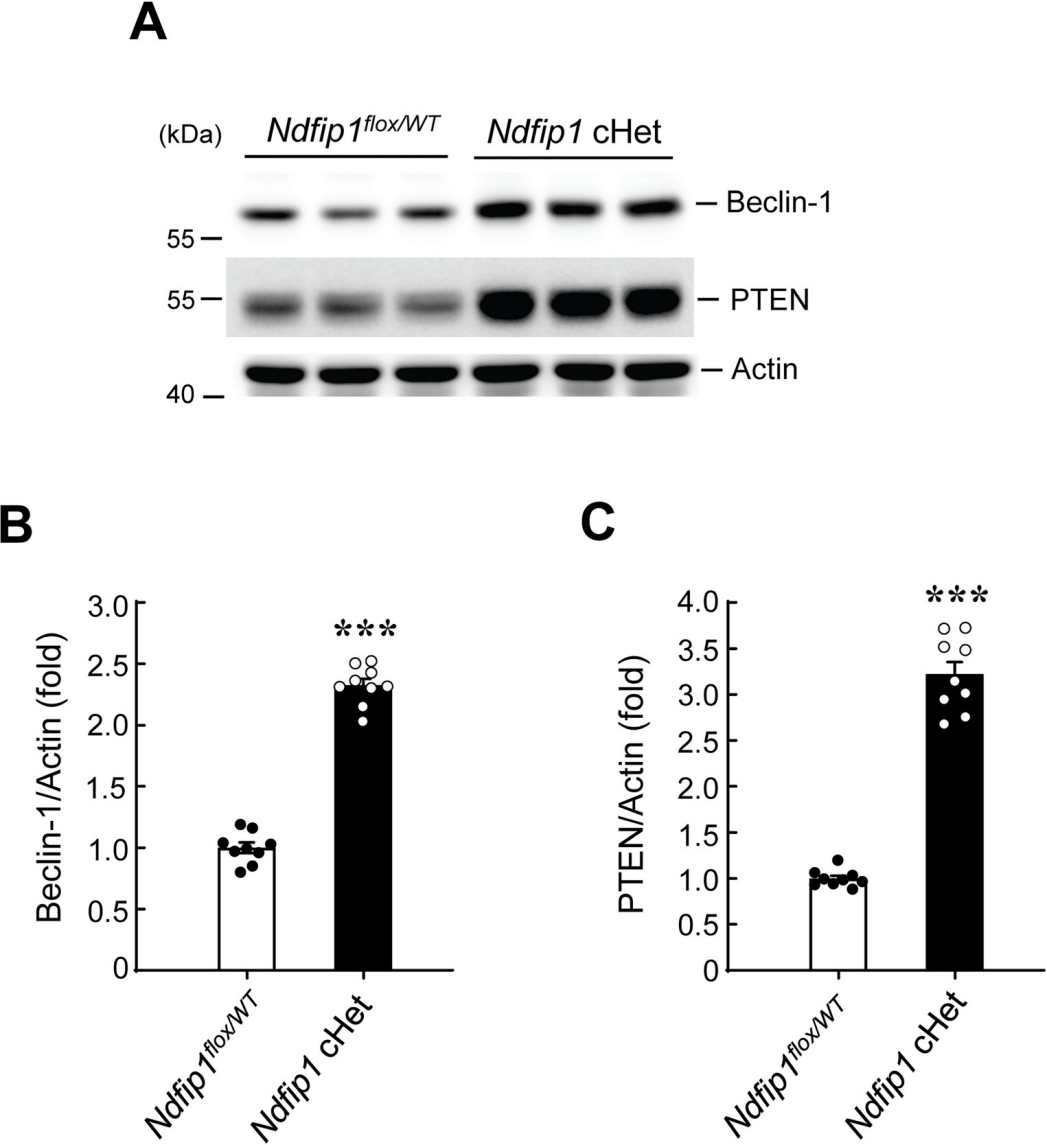
Beclin 1 expression and PTEN expression are increased in *Ndfip1* cHet mice. **(A)** The CA1 tissue lysates from animals in Fig 2 (D-G) were subjected to western blot determination of Beclin 1 and PTEN expression. Both Beclin 1 and PTEN expression level is increased in *Ndfip1* cHet mice than *Ndfip1*^flox/WT^ mice. **(B)** The quantified result of Beclin 1 expression in *Ndfip1*^flox/WT^ mice and *Ndfip1* cHet mice (N = 9 each group) (t_1,16_ = 19.61, *p* < 0.001). **(C)** The quantified result of PTEN expression in *Ndfip1*^flox/WT^ mice and *Ndfip1* cHet mice (N = 9 each group) (t_1,16_ = 16.17, *p* < 0.001). Data are expressed as individual values and mean ± SEM. *** *p* < 0.001.

The above results together showed that spatial learning and memory performance is enhanced in *Ndfip1* cHet mice whereas it is impaired in *Becn1* cKO and *Pten* cKO mice. These results also showed that Ndfip1 promotes Nedd4-mediated ubiquitination of Beclin 1 and PTEN. Although we have shown that spatial training decreased endogenous Beclin 1 and PTEN ubiquitination in the hippocampus, it is not known whether Beclin 1 and PTEN are downstream effectors of Ndfip1 in Ndfip1-mediated memory impairment. To examine this issue, the CA1 tissue lysates from *Ndfip1*^*flox/WT*^ control mice and *Ndfip1* cHet mice that have been subjected to water maze learning (in Fig 2) were subjected to western blot determination of Beclin 1 and PTEN expression. Results revealed that both Beclin 1 expression level (Fig 6A and 6B) and PTEN expression level (Fig 6A and 6C) are significantly increased in *Ndfip1* cHet mice compared to *Ndfip1*^*flox/WT*^ mice.

## Discussion

In the present study, we have found that the *Ndfip1* cDNA fragment is differentially expressed between the fast learners and the slow learners from the water maze learning task with the fast learners showing a lower expression level. Spatial training decreases *Ndfip1* mRNA and protein expression in the hippocampus, whereas the *Ndfip1* cHet mice show improved acquisition and retention performance. Both Beclin 1 and PTEN are endogenous ubiquitination targets of Nedd4 in the hippocampus. Spatial training decreases the association between Ndfip1 and Nedd4; meanwhile, it decreases endogenous Beclin 1 and PTEN ubiquitination in the hippocampus. Consistent with these observations, spatial training increases the expression level of Beclin 1 and PTEN, but *Becn1* cKO and *Pten* cKO mice both show impaired spatial learning and memory. Further, Beclin 1 and PTEN expression level is increased in the *Ndfip1* cHet mice compared to the *Ndfip1*^*flox/WT*^ control mice.

Ubiquitination is an important post-translational modification that regulates various cellular processes and physiological functions. The Nedd4 family E3 ubiquitin ligase is one of the WW-HECT domain E3 ubiquitin ligases that recognize the PY motif on other proteins [33]. Ndfip1 is a PY-containing protein and is an adaptor protein for Nedd4 family proteins. Thus, Ndfip1 is believed to promote Nedd4-mediated ubiquitination. In the present study, we have found that the association between Ndfip1 and Nedd4 is decreased in the hippocampus of animals subjected to spatial training. It is conceivable that Ndfip1 activation of Nedd4 is also decreased in the hippocampus of trained animals. This speculation is partly supported by our findings that *Ndfip1* siRNA decreased Nedd4-mediated ubiquitination of Beclin 1 and PTEN in the cell and that spatial training decreased endogenous Beclin 1 and PTEN ubiquitination in the hippocampus. It is also supported by our observations that Beclin 1 expression and PTEN expression are both increased in *Ndfip1* cHet mice which showed enhanced spatial learning and memory performance. Although we have shown that Beclin 1 and PTEN are both the endogenous ubiquitination targets of Nedd4 in the hippocampus, our results do not exclude the possibility that Beclin 1 and PTEN are also the ubiquitination targets of other Nedd family proteins involved in Ndfip1-mediated memory impairment.

Other than its role in the inhibition of tumorigenesis, Beclin 1 and post-translational modifications of Beclin 1 are well documented to play a role in autophagy regulation [24,34,35]. On the other hand, autophagy in the hippocampus is suggested to be required for memory formation in mice [36] and that inhibitory avoidance learning was shown to increase the levels of autophagy and a few lysosomal degradation proteins, including Beclin 1, in the rat hippocampus [25]. These results support our findings that Beclin 1 expression in the hippocampus is increased in animals subjected to water maze learning and that *Becn1* cKO mice show impaired spatial learning and memory performance. But we further indicate that training-induced Beclin 1 expression is likely due to decreased Beclin 1 ubiquitination by spatial training. Our results are also congruent with the role of Beclin 1 found in pathological memory [37]. For example, Beclin 1 expression level was found decreased in the brain of Alzheimer’s disease (AD) patients whereas overexpression of Beclin 1 reduces the accumulation of amyloid-beta in an animal model of AD [38]. But few opposite results were also reported. For example, deficiency of activated C kinase was found to impair memory formation associated with upregulation of Beclin 1 [39]. The reason for this discrepancy requires clarification.

PTEN is well known for its role as a tumor suppressor and *Pten* mutations were frequently found in various human cancers [40]. PTEN signaling also plays an important role in some neuronal functions and brain diseases [41]. In the present study, we have found that PTEN expression is important for spatial memory formation. Our results are consistent with the findings that PTEN deletion causes deficits in fear conditioning learning [42] and that spatial learning and LTP are impaired in PTENα-deficient mice [28]. Our results are also congruent with the reports that *Pten* cKO mice show dysregulated synaptic plasticity [27]. But we have provided novel mechanism that downregulation of PTEN expression is a downstream event of Ndfip1 signaling in association with Nedd4, and Ndfip1 expression is negatively regulated by spatial training. Further, we have found that spatial training decreased endogenous PTEN ubiquitination in the hippocampus. However, the present results and results from the above studies are incongruent with the observation that PTEN inhibition rescues cognitive impairment in APP/PS1 mice [43]. A possible explanation for this discrepancy is that for the latter study, the pharmacological tool adopted is targeted specifically at the PTEN and PDZ motif-dependent interactions, whereas for the study of Wang et al. [28], it is emphasized on the PTEN-CaMKII-NMDA receptor signaling. In addition, PTEN was found to dephosphorylate phosphatidylinositol 3,4,5-trisphosphate (PIP3) and negatively regulate phosphatidylinositol-3-kinase (PI3K) signaling [44], but an early study has shown that PI3K activation is essential for fear memory formation [45]. These results do not conflict each other because PTEN could regulate signaling pathways other than PI3K signaling for memory processing, and the net result is facilitation of memory formation.

The present results show that Ndfip1 impairs spatial learning and memory and this is probably mediated through Nedd4-mediated ubiquitination of Beclin 1 and PTEN. These results implicate that Ndfip1 negatively regulates neuronal plasticity, but they are inconsistent with the observations that Ndfip1 is required for the development of neuronal dendrites and spines [46] and Ndfip1 is associated with neuronal survival upon brain injury [22]. Our results are also incongruent with the report that decreased Ndfip1 expression is associated with AD pathogenesis [47]. We do not know the explanations for these discrepancies yet. It is possible that Ndfip1 interacts with different Nedd4 family proteins in these studies or that Ndfip1 may play different roles in different physiological and pathological conditions. It is also possible that Ndfip1 impairs learning and memory through Nedd4-mediated ubiquitination of other proteins in addition to Beclin 1 and PTEN. For example, alterations of AMPA receptor surface expression, trafficking and turnover are important mechanisms underlying synaptic plasticity [48,49], and AMPA receptor was found as an ubiquitination substrate of Nedd4 and AMPA receptor ubiquitination by Nedd4 results in decreased AMPA receptor surface expression and decreased excitatory synaptic transmission [50]. Further, we have previously shown that SGK expression facilitates spatial learning and memory formation in rats [9]. SGK was ubiquitinated and degraded by Nedd4-2 [51] and that in part accounts for the low endogenous expression level of SGK [52]. Based on these findings, it is reasonable to expect that downregulation of Ndfip1 by spatial training stabilizes SGK expression and facilitates memory formation. Moreover, Ndfip1 was found to recruit Nedd4-2 and mediates the ubiquitination of TrkB, a neurotrophin receptor that mediates brain-derived neurotrophic factor (BDNF) signaling [53], and conditional deletion of *Ndfip1* increases TrkB expression in the hippocampus [54], whereas BDNF plays a critical role in mammalian learning and memory formation [55,56]. Because Ndfip1 is the adaptor protein for both Nedd4 (Nedd4-1) and Nedd4-2 [57], it is likely that more Nedd4 family substrate proteins are involved in spatial learning and memory formation through the regulation by Ndfip1. On the other hand, another study has shown that spatial memory and LTP are both impaired in Nedd4 heterozygous mice [58]. We do not know the explanation for the discrepancy between this study and our study as well as the above studies. It is possible that the effect of whole brain Nedd4 reduction is different from that of hippocampal Nedd4 reduction in terms of spatial learning and memory processing. It is also possible that compensation mechanism may occur to Nedd4-2 that takes place the function of Nedd4 (Nedd4-1) due to reduced Nedd4 level in the brain. In the present study, we have adopted the *Ndfip1*^*flox/WT*^ genotype of mice for experimentation. The reason is that most of the mice we obtained are the *Ndfip1*^*flox/WT*^ mice and only few *Ndfip1*^*flox/flox*^ mice were generated through inbreeding. In addition, the body size of the *Ndfip1*^*flox/flox*^ mice was also smaller. We do not know the reason behind these phenomena, but it is not because DNA insertion of the loxP cassette prevents Ndfip1 protein expression because the Ndfip1 expression level is similar between the wild-type mice and the *Ndfip1*^*flox/WT*^ mice (S5 Fig).

In this study, the slow learners and non-trained rats both show a higher level of Ndfip1 expression. A common mechanism may be due to the relatively low level of NMDA receptor activation because we have shown that NMDA administration downregulates Ndfip1 expression (S1 Fig). But the slow learners still gradually learn the task compared to the non-trained rats. There is a possibility that some neurophysiological mechanisms, such as CA1 neuron field potential, might be different in these two groups of mice. Moreover, although we have found that spatial learning and memory is impaired in the *Ndfip1* cHet mice compared to the *Ndfip1*^*flox/WT*^ mice, the cellular and physiological mechanisms underlying Ndfip1-regulated memory impairment is still not known. It could be possible that synaptic plasticity and CA1 neuron field potential is increased in *Ndfip1* cHet mice. It is also likely that Ndfip1 may indirectly downregulate the expression of certain proteins that are known to facilitate learning and memory formation, such as BDNF. The physiological role of Ndfip1 in negative regulation of memory formation requires further investigation. The same issue applies to the *Becn1* cKO mice and *Pten* cKO mice, but the cellular mechanisms could be different from that of the *Ndfip1* cHet mice.

**Fig 7:**
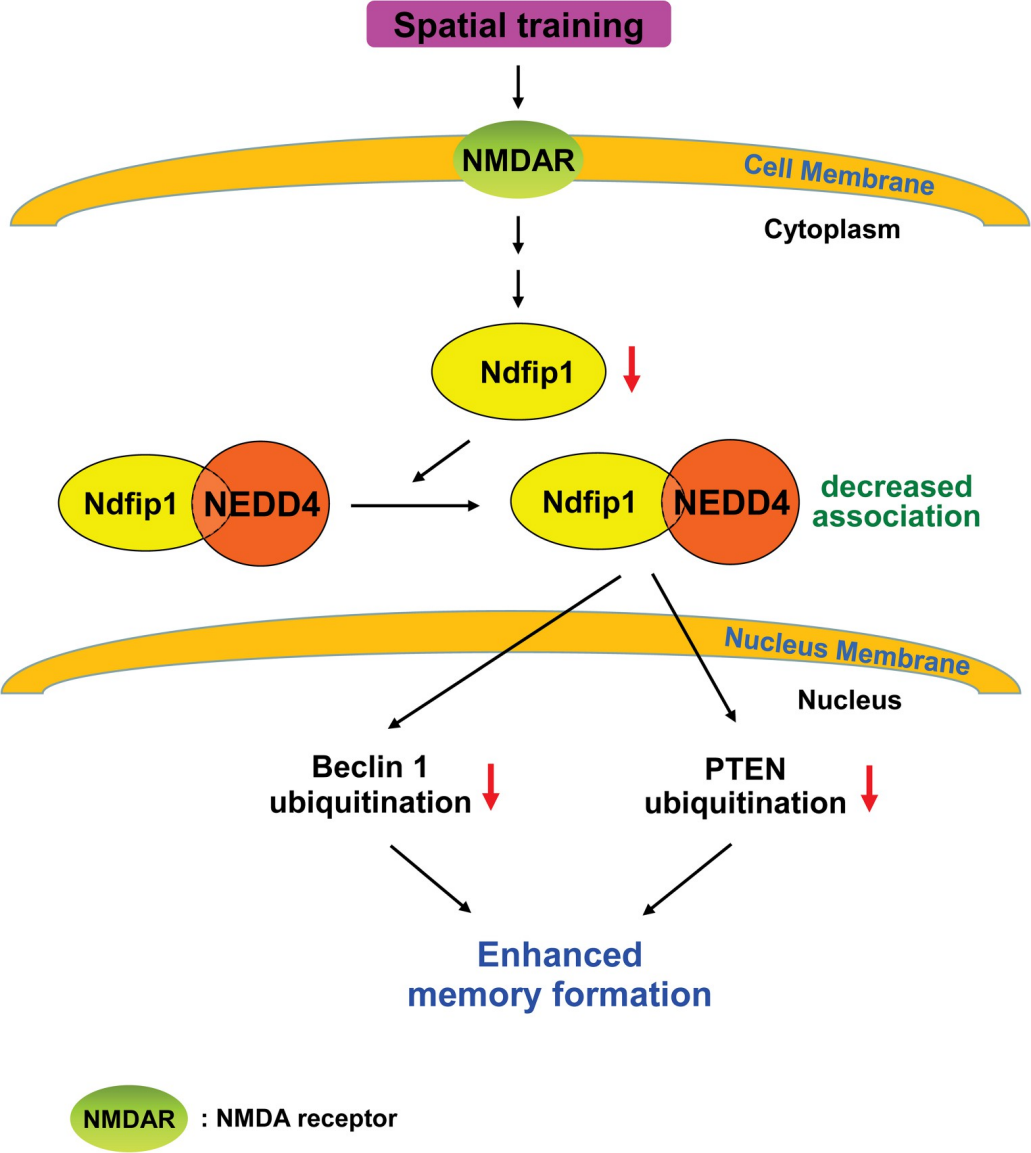
An illustration showing that spatial training induces decrease of Ndfip1 expression through NMDA receptor mediation, and it subsequently reduces the association between Ndfip1 and Nedd4, that results in decreased Beclin 1 and PTEN ubiquitination and enhanced spatial memory formation.

In summary, we have shown that *Ndfip1* might be an important candidate gene in negative regulation of spatial memory formation, and this is associated with its interaction with Nedd4 and increased ubiquitination of Beclin 1 and PTEN in the hippocampus (Fig 7). Our results are consistent with the notion that protein degradation, other than protein synthesis, also underlies the mechanism of memory formation, but the physiological mechanisms underlying Ndfip1-regulated memory impairment remains to be elucidated. In addition, a previous study has shown that Ndfip1 is associated with AD [47]. Here, we have shown that Ndfip1 is involved in negative regulation of memory formation. In future studies, it is worth to examine the role and mechanism of Ndfip1 possibly involved in the pathogenesis or neuroprotection against AD. It is also worth to explore whether Ndfip1 may play a role in neurological disorders that are related to cognitive impairment, such as Rett syndrome. These studies might provide novel therapeutic implication of cognitive impairment associated with these diseases.

## Supporting information

S1 Fig Acute NMDA administration decreases Ndfip1 expression in rat hippocampus. (TIF)

S2 Fig The swim speed for probe trial performance and visible platform learning performance of three genotypes of mice. (TIF)

S3 Fig Beclin 1 is an ubiquitination target of Nedd4 and Beclin 1 ubiquitination is decreased by *Ndfip1* siRNA in HEK293T cells. (TIF)

S4 Fig PTEN is an ubiquitination target of Nedd4 and PTEN ubiquitination is decreased by *Ndfip1* siRNA in HEK293T cells. (TIF)

S5 Fig Ndfip1 expression level is not different between wild-type (WT) mice and *Ndfip1^flox/WT^* mice. (TIF)

S6 Fig (PDF)
