# Supplementary figures and images for: Identification of Ndfip1 as a novel negative regulator for spatial memory formation associated with increased ubiquitination of Beclin 1 and PTEN
Source: PLoS One. 2023 Apr 6;18(4):e0283908. doi: 10.1371/journal.pone.0283908 (PMC10079018; doi:10.1371/journal.pone.0283908)

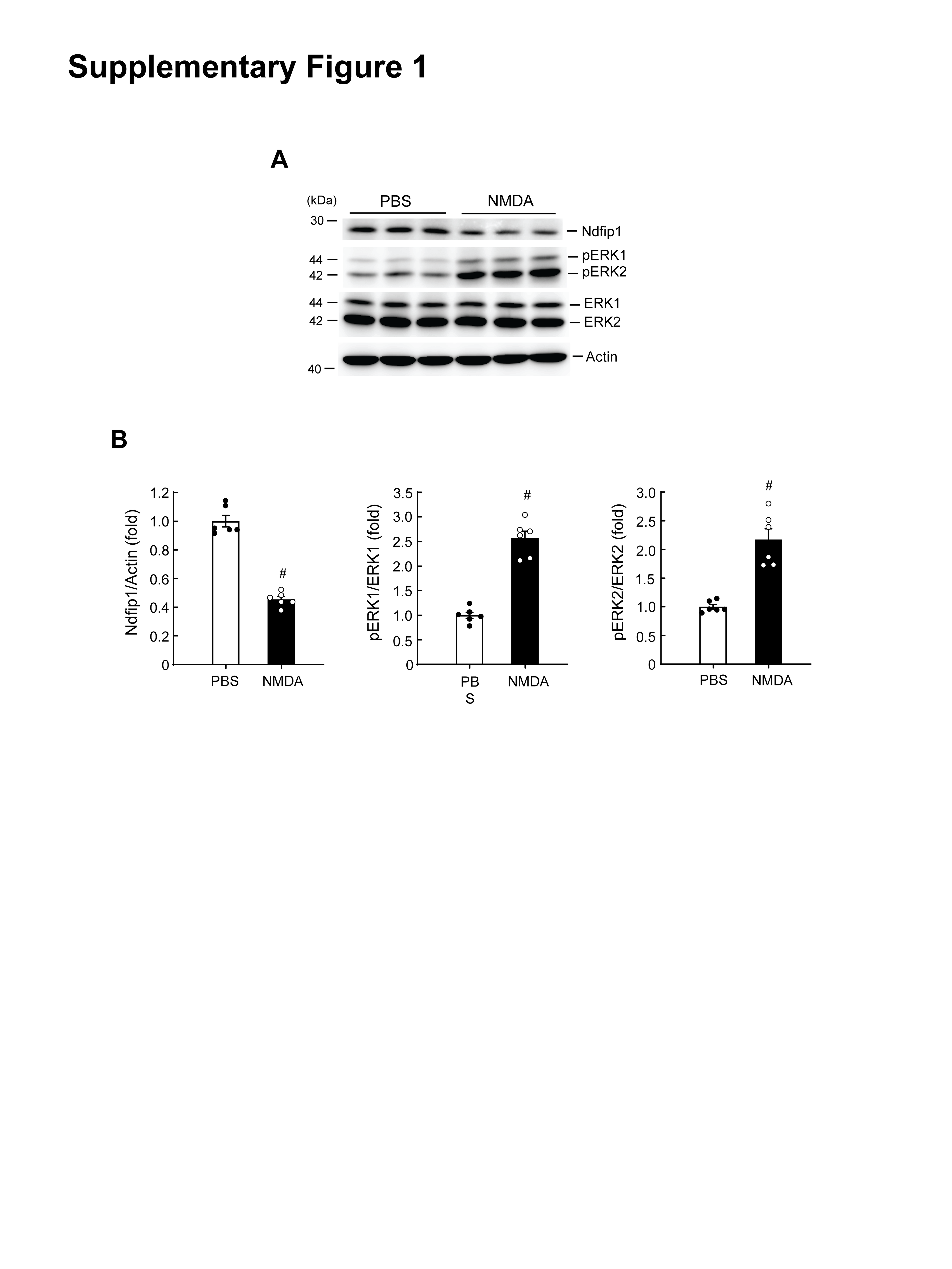

Supplement: S1 Fig — (TIF) [file pone.0283908.s001.tif]

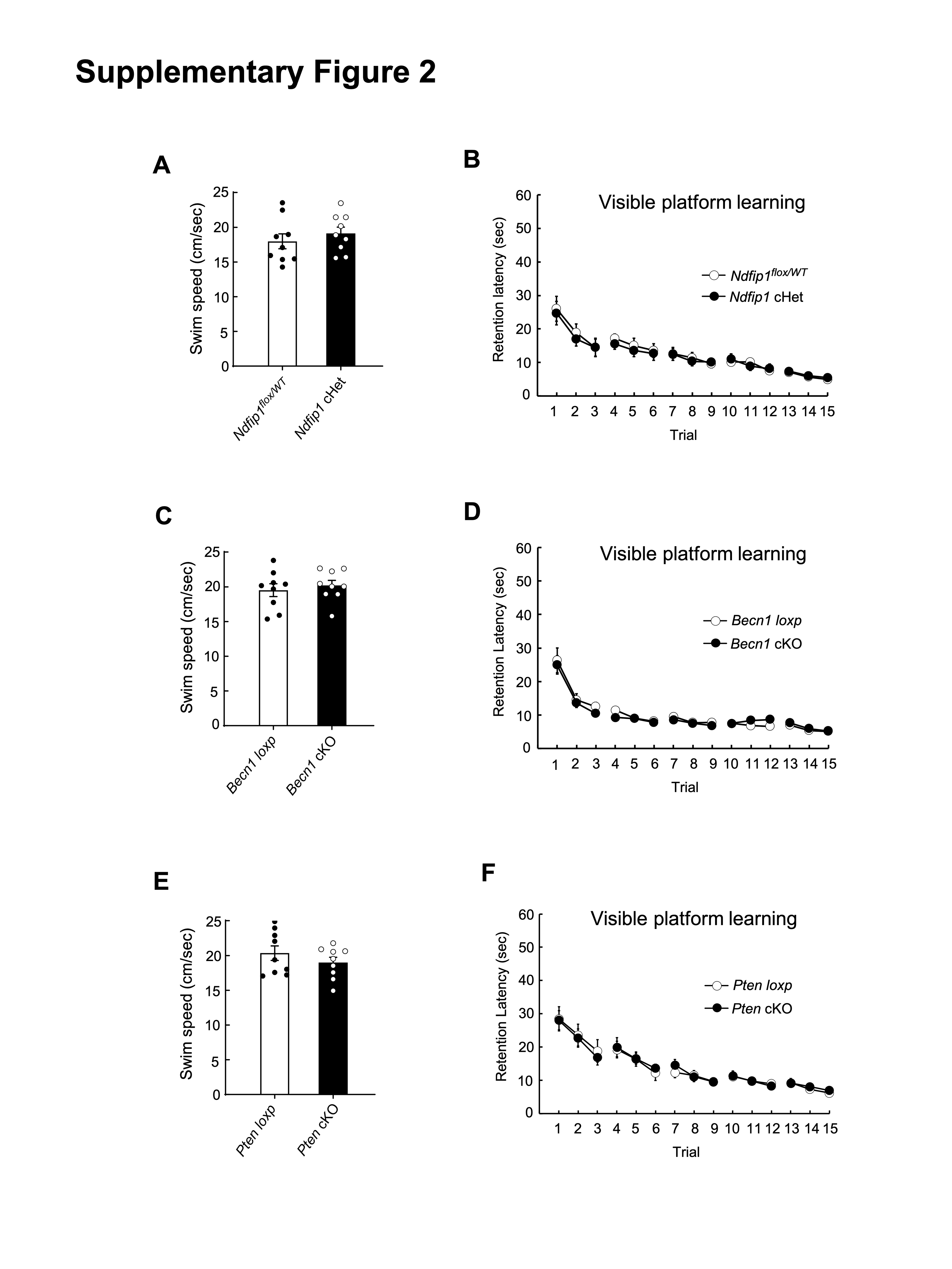

Supplement: S2 Fig — (TIF) [file pone.0283908.s002.tif]

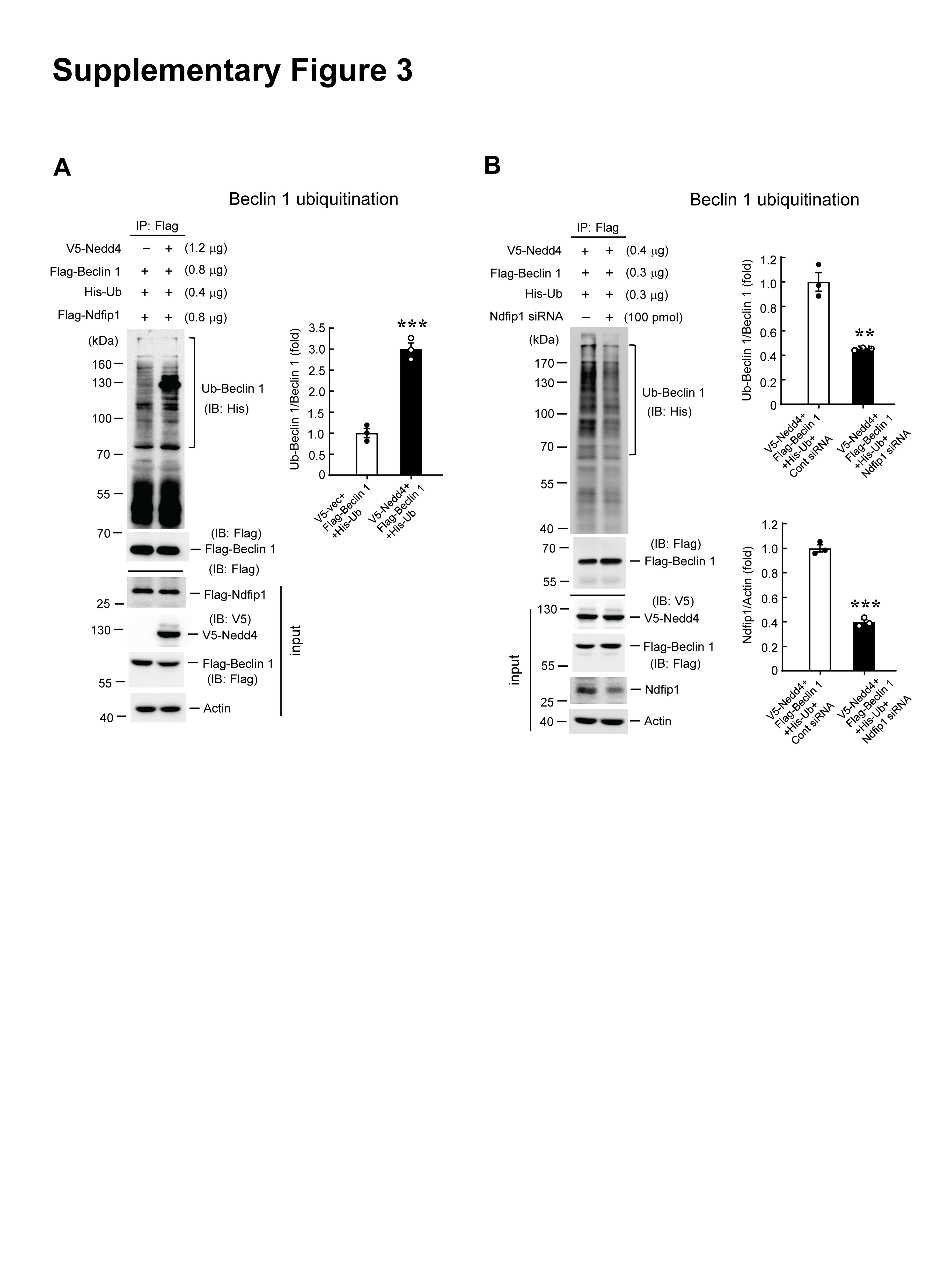

Supplement: S3 Fig — (TIF) [file pone.0283908.s003.tif]

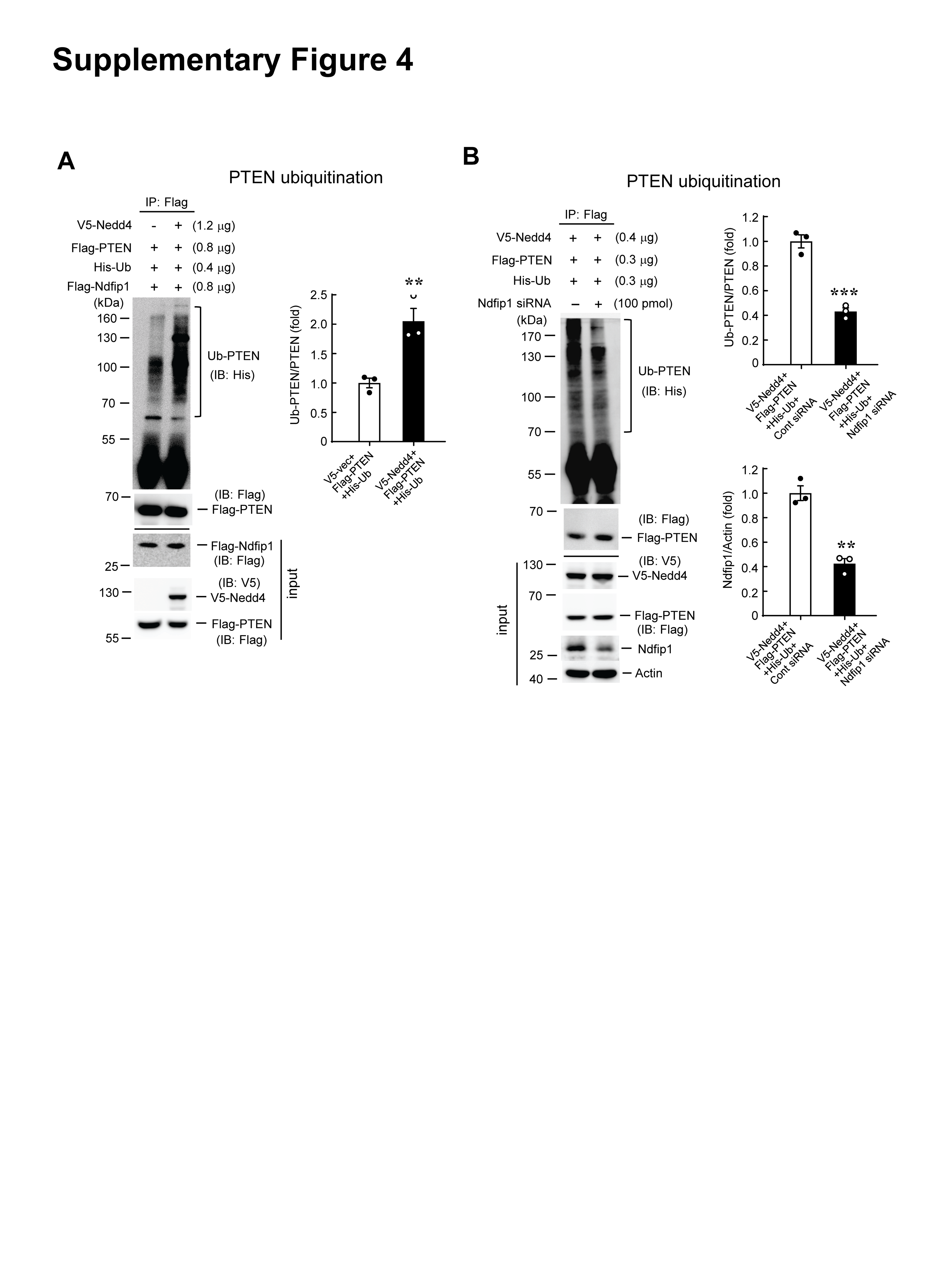

Supplement: S4 Fig — (TIF) [file pone.0283908.s004.tif]

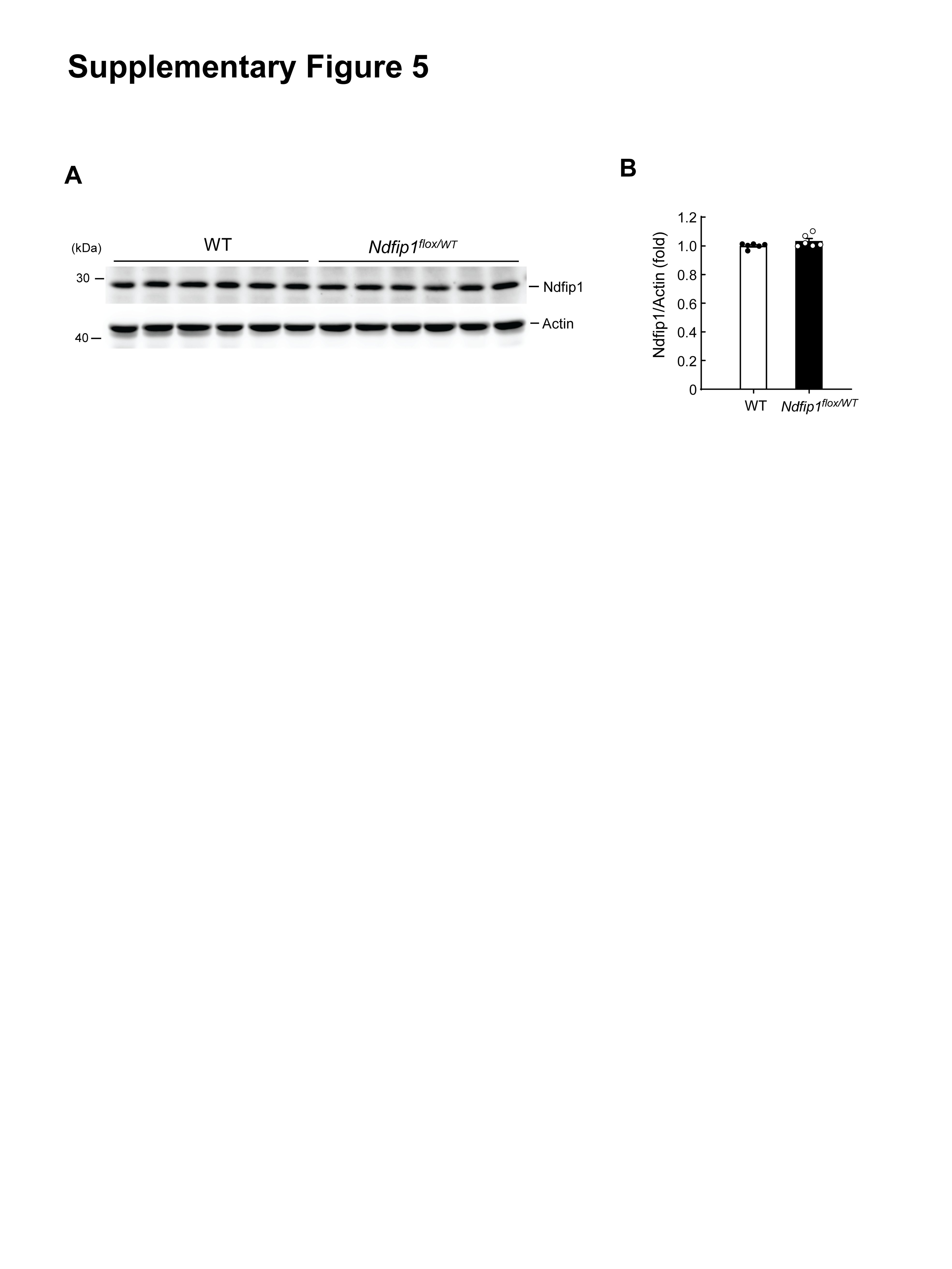

Supplement: S5 Fig — (TIF) [file pone.0283908.s005.tif]
