# Supplementary material for: Identification of Ndfip1 as a novel negative regulator for spatial memory formation associated with increased ubiquitination of Beclin 1 and PTEN
Source: PLoS One. 2023 Apr 6;18(4):e0283908. doi: 10.1371/journal.pone.0283908 (PMC10079018; doi:10.1371/journal.pone.0283908)

Supplementary Figure 6 (original blots for all figures)

Figure 1

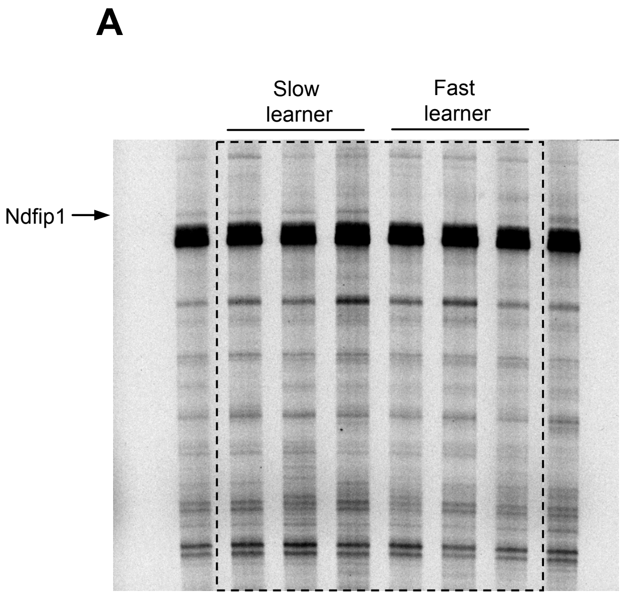

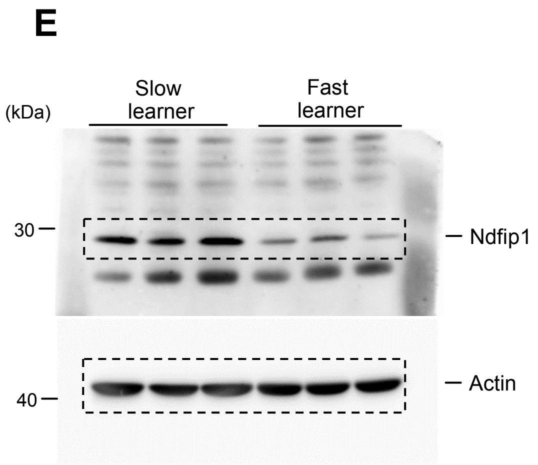

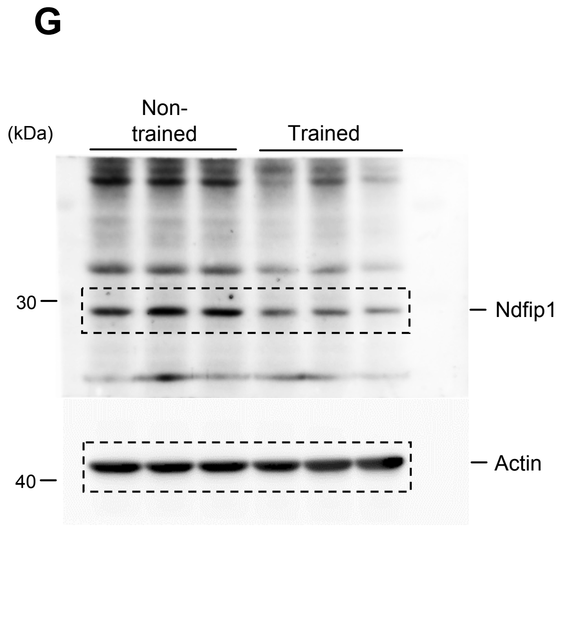

Figure 2

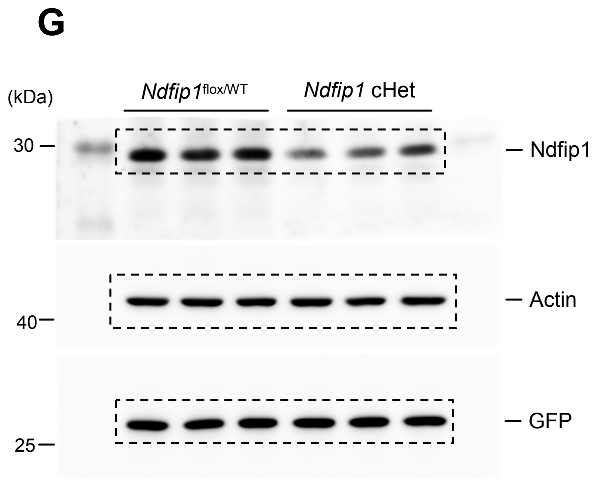

Figure 3

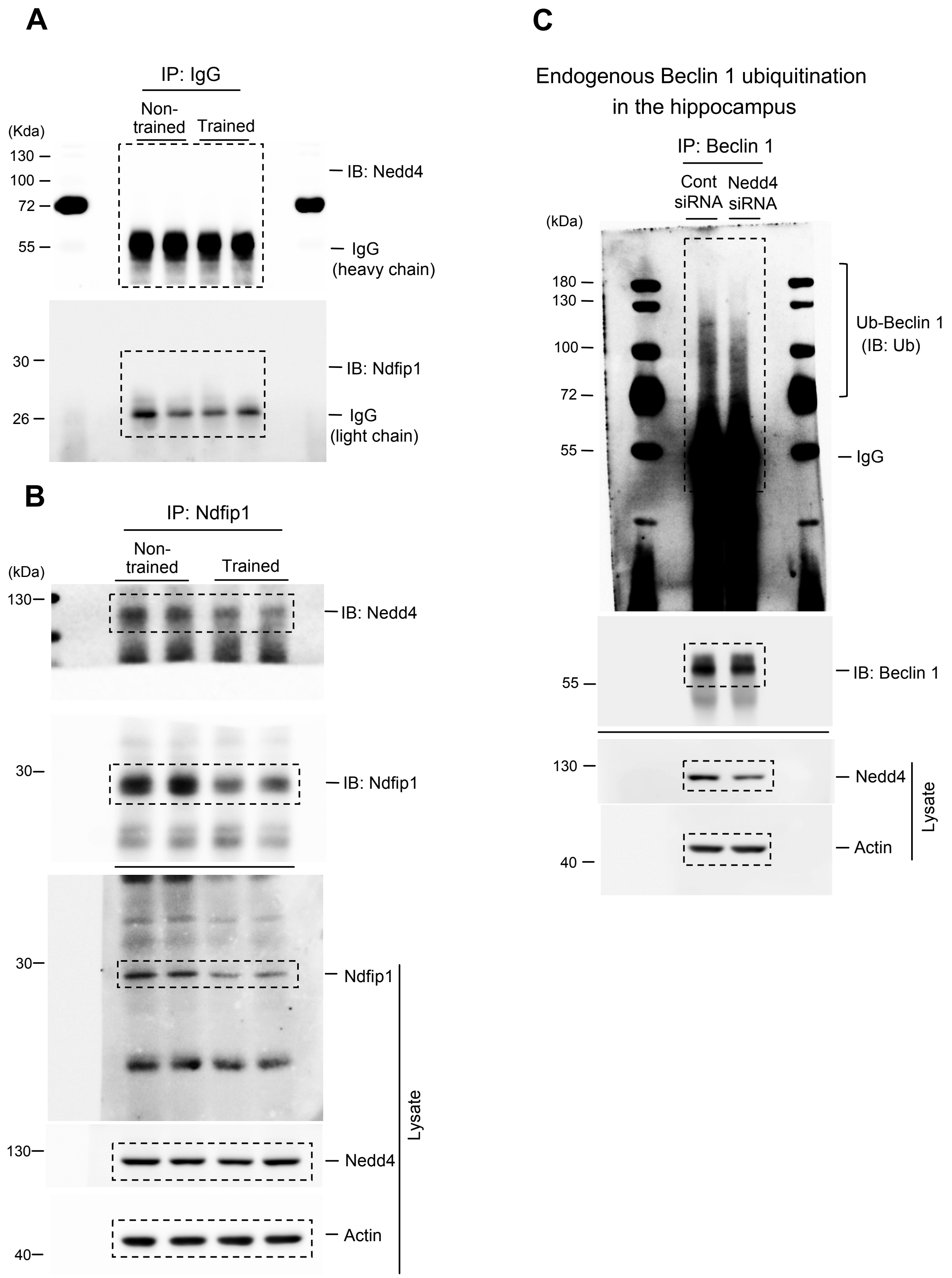
A
IP: IgG
Non-
trained
Trained
(Kda)
130
100
72
55
IB: Nedd4
IgG
(heavy chain)
30
26
IB: Ndfip1
IgG
(light chain)
B
IP: Ndfip1
Non-
trained
Trained
(kDa)
130
IB: Nedd4
30
IB: Ndfip1
30
Ndfip1
130
Nedd4
40
Actin
Lysate
C
Endogenous Beclin 1 ubiquitination
in the hippocampus
IP: Beclin 1
Cont
siRNA
Nedd4
siRNA
(kDa)
180
130
100
72
55
Ub-Beclin 1
(IB: Ub)
IgG
55
IB: Beclin 1
130
Nedd4
40
Actin
Lysate

Figure 3 (continue)

D

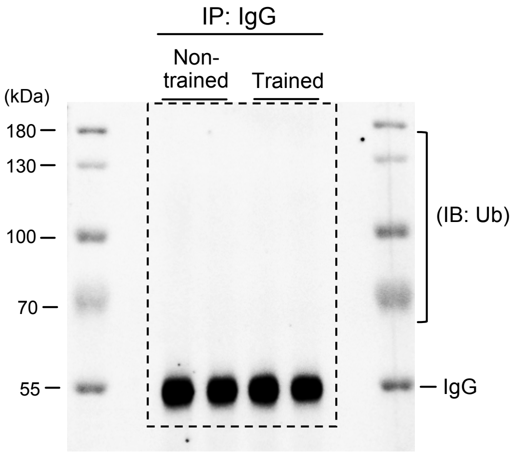

E

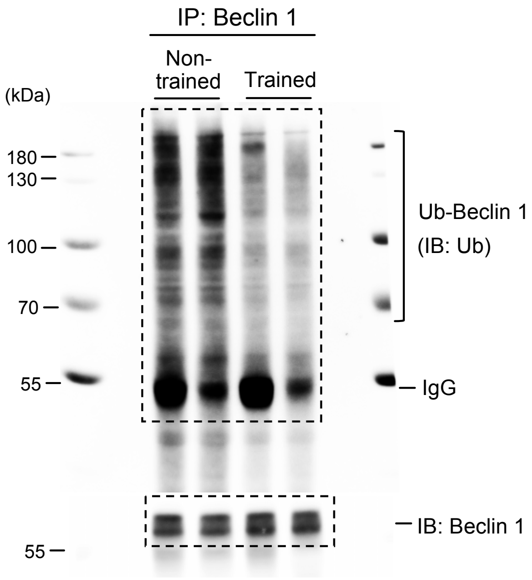

Figure 4

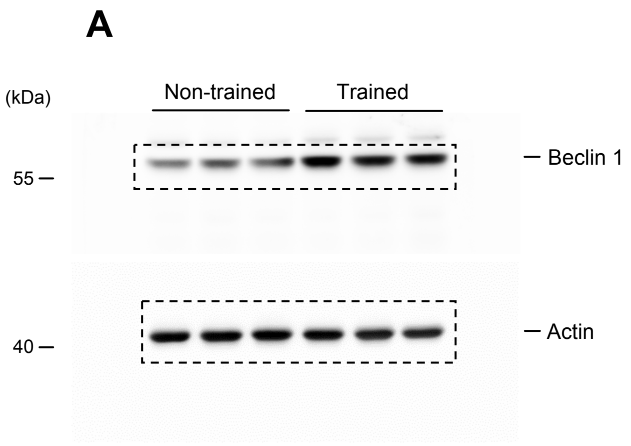
A
(kDa)
Non-trained
Trained
55
Beclin 1
40
Actin

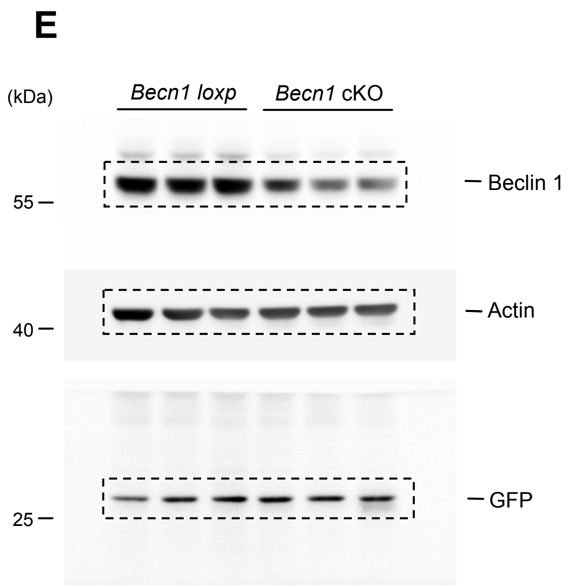
E
(kDa)
Becn1 loxp
Becn1 cKO
55
Beclin 1
40
Actin
25
GFP

Figure 5

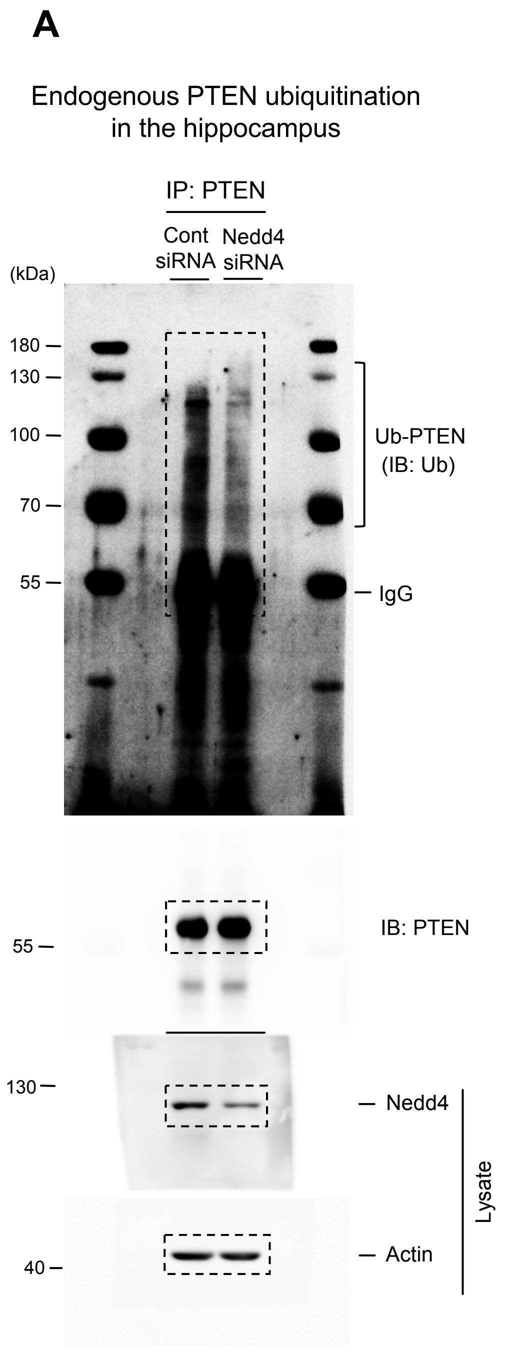
A
Endogenous PTEN ubiquitination
in the hippocampus
IP: PTEN
Cont siRNA
Nedd4 siRNA
(kDa)
180
130
100
70
55
Ub-PTEN
(IB: Ub)
IgG
55
IB: PTEN
130
Nedd4
40
Actin
Lysate

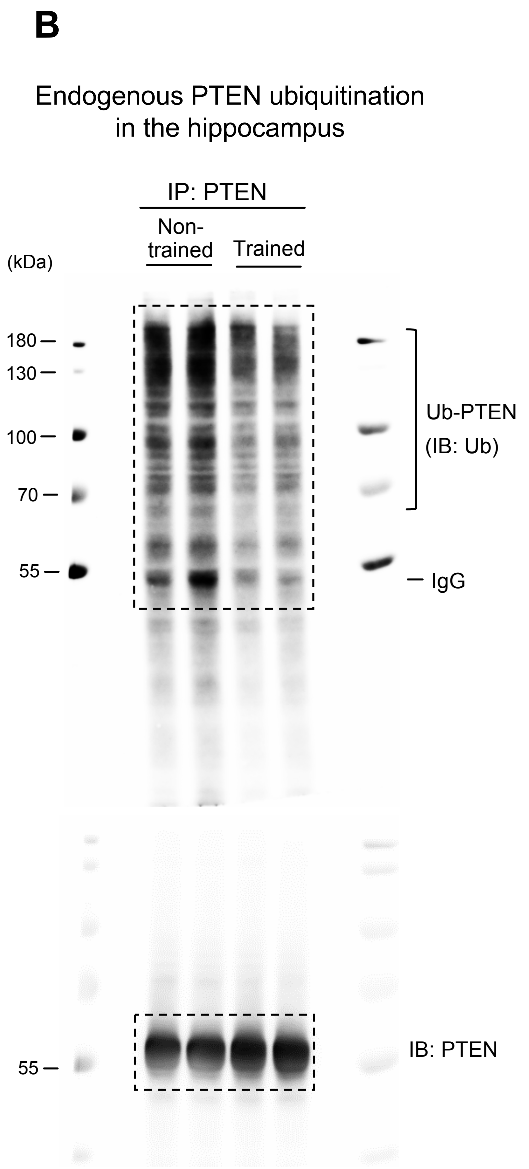
B
Endogenous PTEN ubiquitination
in the hippocampus
IP: PTEN
Non-trained
Trained
(kDa)
180
130
100
70
55
Ub-PTEN
(IB: Ub)
IgG
55
IB: PTEN

Figure 5 (continue)

C

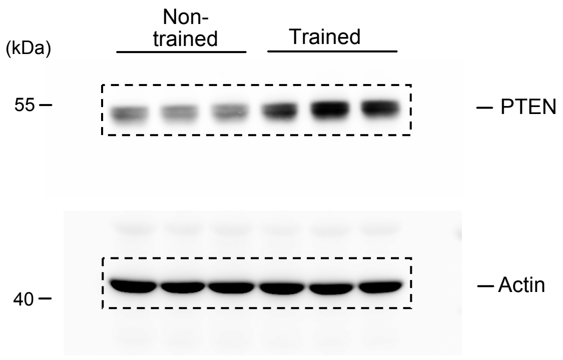

G

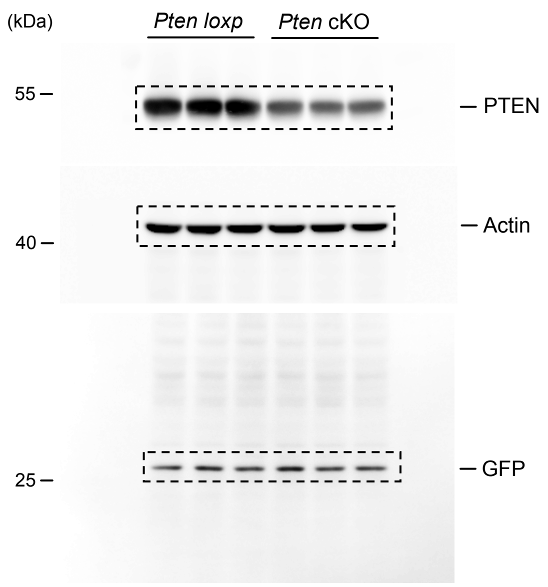

Figure 6

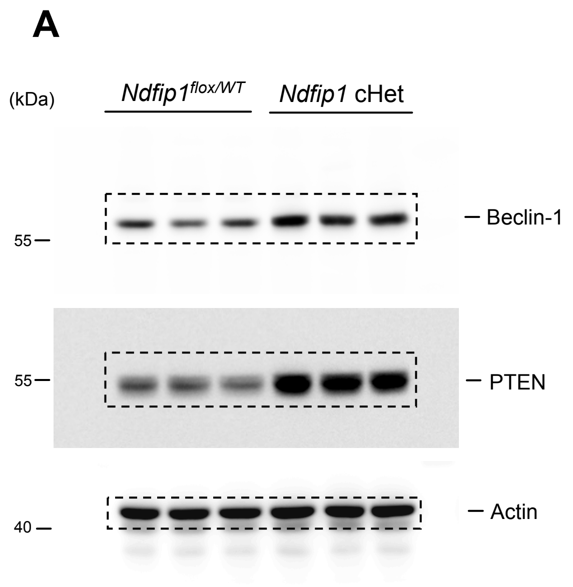

Supplementary Figure 1

A

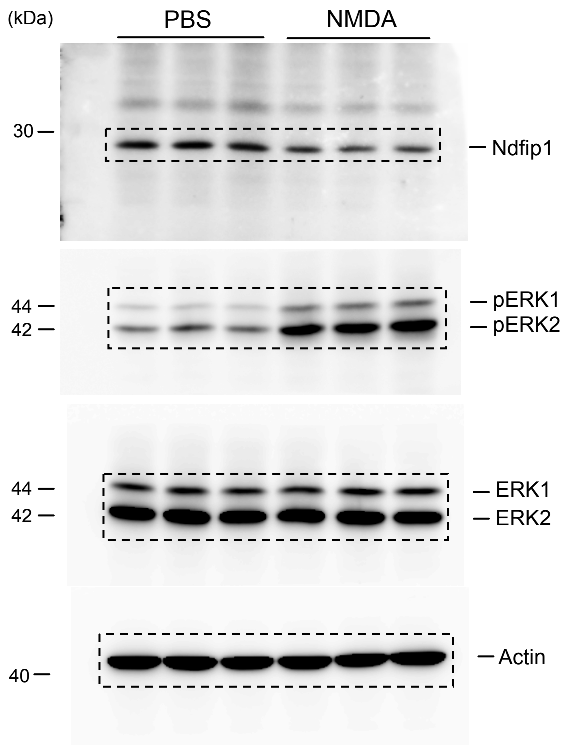

Supplementary Figure 3

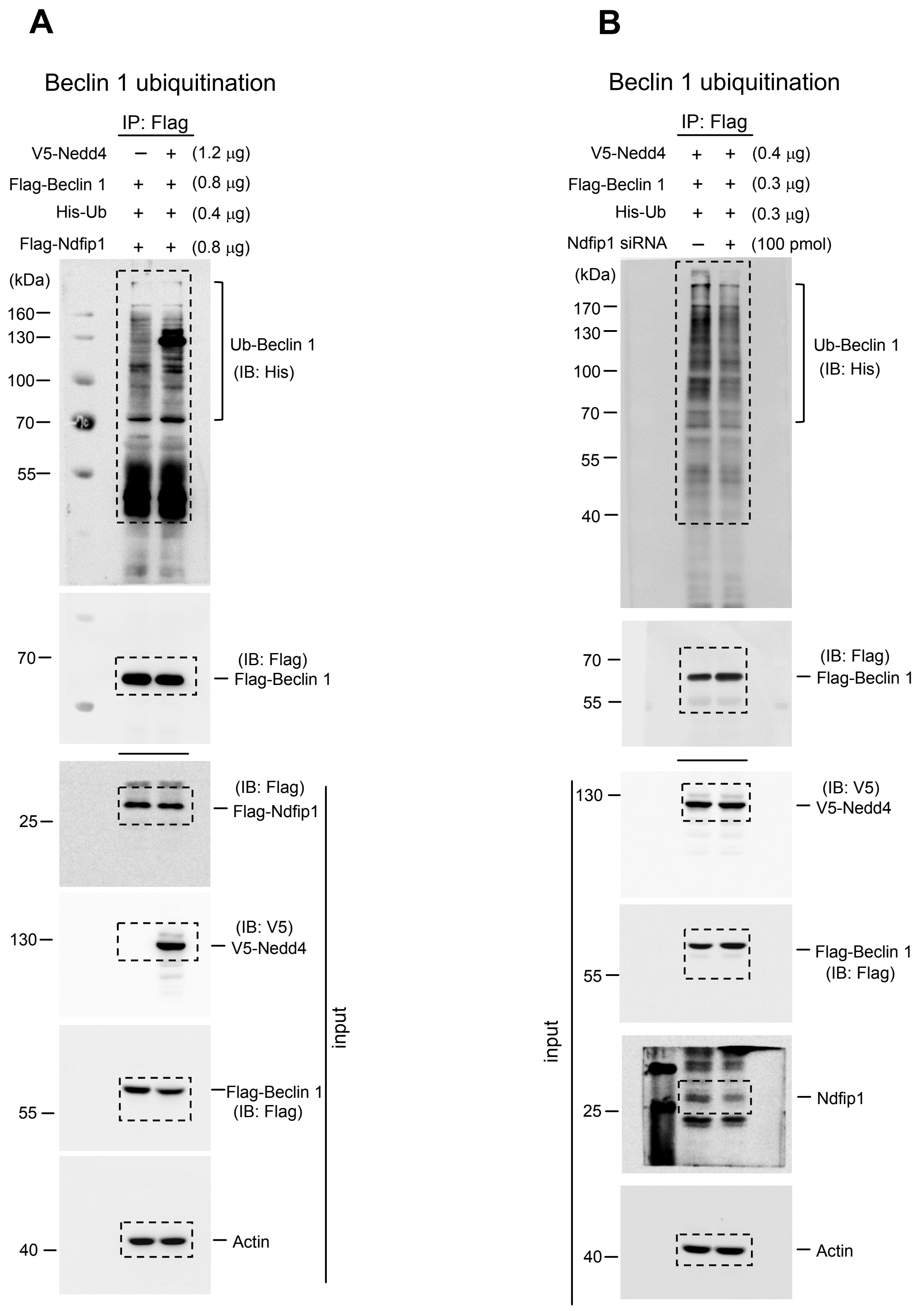

Supplementary Figure 4

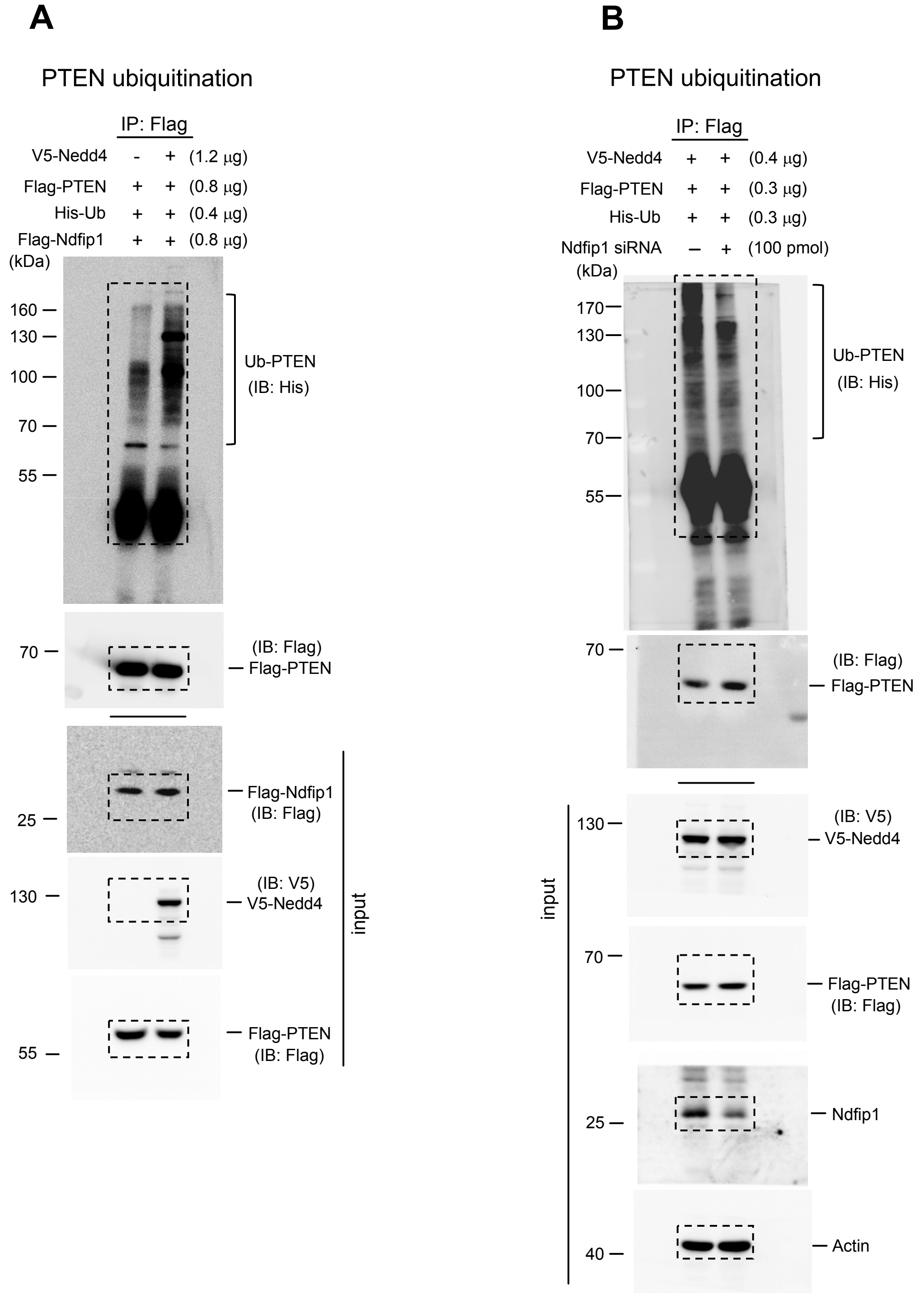

Supplementary Figure 5

A

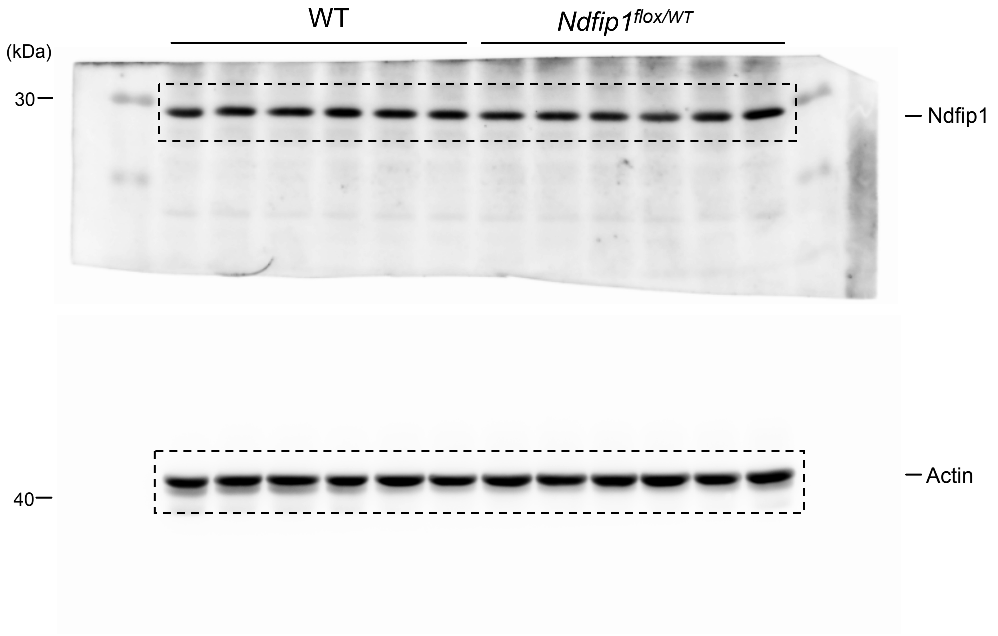

Supplement: S6 Fig — (PDF) [file pone.0283908.s006.pdf]
